# Transparent reporting of multivariable prediction models developed or validated using clustered data: TRIPOD-Cluster checklist

**DOI:** 10.1136/bmj-2022-071018

**Published:** 2023-02-07

**Authors:** Thomas P A Debray, Gary S Collins, Richard D Riley, Kym I E Snell, Ben Van Calster, Johannes B Reitsma, Karel G M Moons

**Affiliations:** 1 Julius Centre for Health Sciences and Primary Care, University Medical Centre Utrecht, Utrecht University, Utrecht, Netherlands; 2 Cochrane Netherlands, University Medical Centre Utrecht, Utrecht University, Utrecht, Netherlands; 3 Centre for Statistics in Medicine, Nuffield Department of Orthopaedics, Rheumatology and Musculoskeletal Sciences, Botnar Research Centre, University of Oxford, Oxford, UK; 4 NIHR Oxford Biomedical Research Centre, John Radcliffe Hospital, Oxford, UK; 5 Centre for Prognosis Research, School of Primary, Community and Social Care, Keele University, Keele, UK; 6 Department of Development and Regeneration, KU Leuven, Leuven, Belgium

## Abstract

The increasing availability of large combined datasets (or big data), such as those from electronic health records and from individual participant data meta-analyses, provides new opportunities and challenges for researchers developing and validating (including updating) prediction models. These datasets typically include individuals from multiple clusters (such as multiple centres, geographical locations, or different studies). Accounting for clustering is important to avoid misleading conclusions and enables researchers to explore heterogeneity in prediction model performance across multiple centres, regions, or countries, to better tailor or match them to these different clusters, and thus to develop prediction models that are more generalisable. However, this requires prediction model researchers to adopt more specific design, analysis, and reporting methods than standard prediction model studies that do not have any inherent substantial clustering. Therefore, prediction model studies based on clustered data need to be reported differently so that readers can appraise the study methods and findings, further increasing the use and implementation of such prediction models developed or validated from clustered datasets.

The TRIPOD (transparent reporting of a multivariable prediction model for individual prognosis or diagnosis) statement provides guidance for the reporting of studies developing, validating, or updating a multivariable prediction model. However, the TRIPOD statement does not fully cover the specific methodological and analytical complexity in prediction model studies that are based on (and thus should account for) clustered data. We therefore developed TRIPOD-Cluster, a new reporting checklist for prediction model studies that are based on clustered datasets. Ten new items are introduced and 19 of the original TRIPOD items have been updated to better reflect issues for clustering. The rationale for each item is discussed, along with examples of good reporting and why transparent reporting is important. TRIPOD-Cluster is best used in conjunction with the TRIPOD-Cluster explanation and elaboration document (www.tripod-statement.org).

Prediction models combine a number of characteristics (predictors or covariates) to produce an individual’s probability or risk of currently having (diagnosis) or developing in a certain time period (prognosis) a specific condition or outcome.^1^ ^2^ ^3^ ^4^ ^5^ They are (still) typically derived using multivariable regression techniques,^2^ ^6^ such as logistic regression (for diagnostic and short term binary prognostic outcomes), Cox proportional hazards regression (for long term binary outcomes), linear regression (for continuous outcomes), or multinomial regression (for categorical outcomes). However, they can be and, increasingly, are developed using machine learning algorithms such as neural networks, support vector machines, and random forests.^7^

Prediction models are widely used to support medical practice and diagnostic, prognostic, therapeutic, and preventive decision making.^3^ ^4^ Therefore, prediction models should be developed, validated, and updated or tailored to different settings using appropriate methods and data sources, such that their predictions are sufficiently accurate and supportive for further decision making in the targeted population and setting.^1^ ^2^ ^8^ ^9^ To evaluate whether a prediction model is fit for purpose, to properly assess their quality and any risks of bias, full and transparent reporting of prediction model studies is essential.^10^

The TRIPOD statement was published in 2015 to provide guidance on the reporting of prediction model studies.^11^ ^12^ The TRIPOD statement comprises a checklist of 22 minimum reporting items that should be addressed in all prediction model studies, with translations available in Chinese and Japanese. However, the TRIPOD statement does not cover important items that arise when prediction models are based on clustered datasets.^13^ ^14^ These datasets arise when individual participant data are obtained or combined from multiple groups, centres, settings, regions, countries, or studies (referred to here as clusters). Observations within a cluster tend to be more alike than observations from other clusters, leading to cluster populations that are different but related.

Clustered datasets can, for instance, be obtained by combining individual participant data from multiple studies (clustering by study; table 1), by conducting multicentre studies (clustering by centre), or by retrieving individual participant data from registries or datasets with electronic healthcare records (clustering by healthcare centre or geographical region; table 2). If differences between clusters are left unresolved during prediction model development, they might not perform well when applied in other or new clusters, and therefore have limited generalisability.^29^ ^30^ Similarly, when heterogeneity between clusters is ignored during prediction model validation, estimates of prediction model performance can be highly misleading.^31^ ^32^ ^33^ In clustered or combined datasets, different individuals in the same cluster have been subject to, for example, similar healthcare processes that have also been delivered by the same healthcare providers, and therefore could be more alike than individuals from different clusters. Sometimes, clusters might also differ in participant eligibility criteria or participation, follow-up length, predictor and outcome definitions, and in (the quality of) applied measurement methods. Hence correlation is likely to be present between observations from the same data cluster,^29^ which can lead to differences or heterogeneity between clusters regarding patient characteristics, baseline risk, predictor effects, and outcome occurrence.

**Table 1:** Examples of prediction model studies that are based on individual participant data meta-analysis from various medical domains and settings

IPD-MA project	Population	No of included studies	Total sample size	Type of prediction model study
IPPIC Network	Pregnant women	78	~3.6 million	External validation of previously published prognostic prediction models for developing pre-eclampsia^15^
Emerging Risk Factors Collaboration	People from the general population without known cardiovascular disease	120	~1.8 million	Development and validation of prediction models for predicting 10 year risk of fatal and non-fatal cardiovascular disease^16^ ^17^
ZIKV IPD Consortium	Pregnant women and their infants exposed to the Zika virus	42	~30 000	Development and validation of prognostic models to predict the risk of miscarriage, fetal loss, and microcephaly^18^
Diagnostic individual patient data meta-analysis of published primary studies on the diagnosis of deep vein thrombosis	Outpatients suspected for deep vein thrombosis	13	~10 000	External validation study to assess the accuracy of the Wells rule for excluding deep vein thrombosis across different subgroups of patients^19^
IMPACT database of traumatic brain injury	Patients with severe and moderate brain injuries	11	~9000	Development of prognostic models to predict the risk of six month mortality and unfavourable outcomes^20^ ^21^

IMPACT=International Mission for Prognosis And Clinical Trial; IPD-MA=individual participant data meta-analysis; IPPIC=International Prediction of Pregnancy Complication; ZIKV IPD=Zika virus individual participant data.

**Table 2:** Examples of prediction model studies that are based on electronic healthcare records from various medical domains and settings

EHR database	Population	No of included clusters	Total sample size	Type of prediction model study
QResearch database	Patients visiting a general practitioner	1309 general practices	~10.5 million	Development and validation of the prognostic QRISK3 model to estimate the future risk of cardiovascular disease^22^ ^23^
National ICD Registry	Patients undergoing ICD implantation in routine clinical practice	1428 hospitals	~170 000	Development of a prediction model to estimate the risk for in-hospital adverse events among patients undergoing ICD placement^24^ ^25^
CALIBER	Patients visiting a general practitioner	>300 general practices	~100 000	Development and validation of prognostic models for all-cause mortality and non-fatal myocardial infarction or coronary death in patients with stable coronary artery disease^26^ ^27^
National Vascular Database	Patients undergoing abdominal aortic aneurysm repair	140 hospitals	~11 000	Development of a risk prediction model for elective abdominal aortic aneurysm repair^28^

CALIBER=Cardiovascular disease research using Linked Bespoke studies and Electronic Health Records; EHR=electronic healthcare records; ICD=implantable cardioverter-defibrillators.

The presence of clustering has important benefits for prediction model research because it enables us to explore heterogeneity in prediction model performance (eg, the model’s calibration and discrimination) across, for example, multiple subgroups, centres, regions, or countries. Identifying sources of heterogeneity helps to better tailor or match the models to these different clusters, and thus to develop prediction models that are more generalisable. However, this strategy requires prediction model researchers to adopt more specific methods for design, analysis, and reporting than standard prediction model studies that do not have any inherent substantial clustering. Therefore, prediction model studies based on clustered data need to be reported differently, so that readers can appraise the study methods and findings, further increasing the use and implementation of prediction models developed or validated from clustered datasets. These specifics are not explicitly mentioned in the original TRIPOD statement.^13^ ^30^ ^31^

Similar to how CONSORT Cluster is an extension of CONSORT,^32^ we developed a new standalone prediction model reporting guideline, TRIPOD-Cluster, that provides guidance for transparent reporting of studies that describe the development or validation (including updating) of a diagnostic or prognostic prediction model using clustered data. TRIPOD-Cluster focuses on clustered studies as present in individual participant data meta-analysis (IPD-MA) and electronic healthcare records datasets (table 1 and table 2), and not on studies with clustering defined by repeated measurements within the same individuals. TRIPOD-Cluster is best used in conjunction with the TRIPOD-Cluster explanation and elaboration document. To aid the editorial process and readability of prediction model studies based on clustered data, it is recommended that authors include a completed checklist in their submission. All information is available online (www.tripod-statement.org).

**Summary points**

To evaluate whether a prediction model is fit for purpose, and to properly assess their quality and any risks of bias, full and transparent reporting of prediction model studies is essential
The TRIPOD-Cluster statement is a new reporting checklist for prediction model studies that are based on clustered datasets
Clustered datasets can be obtained by combining individual participant data from multiple studies, by conducting multicentre studies, or by retrieving individual participant data from registries or datasets with electronic healthcare records
Presence of clustering can lead to differences (or heterogeneity) between clusters regarding participant characteristics, baseline risk, predictor effects, and outcome occurrence
Performance of prediction models can vary across clusters, and thereby affect their generalisability
Additional reporting efforts are needed in clustered data to clarify the identification of eligible data sources, data preparation, risk-of-bias assessment, heterogeneity in prediction model parameters, and heterogeneity in prediction model performance estimates 

## Predictions with clustered data: challenges and opportunities

Overfitting is a common problem in prediction model studies that use a single and often small dataset from a specific setting (eg, a single hospital or practice). Prediction models that are overfitted or overly simple tend to have poor accuracy when applied to new individuals from other hospitals or practices. In addition, when validation studies are also based on single, small, and local datasets, estimates of a prediction model’s performance likely become inaccurate and too optimistic, and fail to show generalisability across multiple medical practices, settings, or countries.^33^ Many examples of prediction models do not perform adequately when applied to new patients.^34^ ^35^ ^36^

To overcome these problems, interest in prediction model studies using large clustered datasets has grown. Combining data from multiple clusters has various advantages:

- Prediction models developed, validated, or updated from larger combined datasets are less prone to overfitting, and estimates of prediction model performance are less likely to be optimistic.
- Researchers can better investigate more complex but more predictive associations, such as including non-linearity of predictor effects, predictor interactions, and time varying effects.
- The presence of multiple large clusters allows development and direct validation of models across a wide range of populations and settings, and thus allows direct tests of a model’s generalisability.^13^ ^37^
- Researchers can better explore and quantify any heterogeneity in a model’s predictive performance across multiple subgroups, centres, regions, or countries to better tailor or match the models to these different clusters, which is desirable when deciding on their use for individualised decision support.^38^

All these benefits of prediction modelling using clustered datasets improve model performance across new clusters, and help develop, validate, and update prediction models that are more generalisable across multiple types of clusters. Prediction model studies using clustered data include important additional details that are not explicitly detailed in the original TRIPOD statement.^11^ ^12^ These details include varying predictor and outcome definitions across clusters, varying (quality of) predictor and outcome measurements, varying inclusion criteria or participation of individuals, the presence and handling of systematically missing data across and within clusters (ie, a predictor not measured for any individuals in a cluster), and varying design related aspects such as blinding. Accordingly, when a clustered dataset is used, special efforts in the development and validation of the prediction model might be needed during the analysis to avoid bias. 

In general, adopting statistical methods that specifically account for clustering is recommended, because the presence of cluster effects could lead to differences in estimated model parameters and in the model’s predictive accuracy across clusters, such that ignoring clustering leads to limited generalisability and applicability.^39^ ^40^ ^41^ ^42^ For instance, random effects models might be needed during prediction model development to allow for heterogeneity across clusters in baseline risk and predictor effects, which yields prediction models with varying intercepts and predictor effects for different clusters.^39^ ^40^ Random effects models might also be needed during model validation to assess heterogeneity in prediction model performance across clusters to gain a deeper understanding of where and in which clusters the model does not satisfy.^37^ Other strategies to account for clustering might consider adjustment for cluster level variables, or implement variable selection algorithms that reduce the presence of cluster heterogeneity in model predictions.^41^ Strategies to explore the presence of clustering and account for statistical heterogeneity across clusters should be adequately reported, and are discussed in the TRIPOD-Cluster explanation and elaboration document.

**Fig 1:**
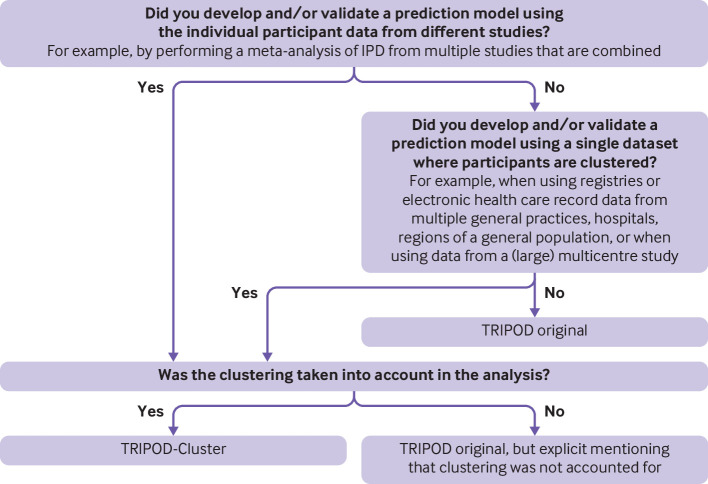
Flow chart to determine whether the published study should follow the original TRIPOD statement or TRIPOD-Cluster checklist

None of the aspects mentioned above is included in the original TRIPOD checklist. For this reason, the present TRIPOD-Cluster checklist provides guidance for the reporting of studies describing the development, validation, and updating of a multivariable diagnostic or prognostic prediction model using clustered data. We provide a flow chart to help authors decide whether to use TRIPOD-Cluster or the original TRIPOD checklist (fig 1). We further refer to the explanation and elaboration document for detailed reasoning and guidance why a new cluster specific item has been added to the original TRIPOD checklist, in which we also use the example studies listed in table 1 and table 2.

## Development of TRIPOD-Cluster

We followed the strategy proposed by the EQUATOR (enhancing the quality and transparency of health research) Network (www.equator-network.org) for developing health research reporting guidelines and formed an executive group (consisting of Doug Altman, GC, TD, KM, JR, and RR).^42^

The first step consisted of reviewing the literature and the EQUATOR Network database (www.equator-network.org) to identify existing reporting guidelines for large (clustered) data or IPD-MA studies in general, including any extensions of these guidelines. Reporting guidelines have been published for various research designs, and these contain many items that are also relevant to prediction model studies using clustered data. In particular, the PRISMA (preferred reporting items for systematic reviews and meta-analyses) checklist of individual participant data (PRISMA-IPD),^43^ STROBE (strengthening the reporting of obeservational studies in epidemiology) statement,^44^ and RECORD (reporting of studies conducted using observational routinely collected data) guideline^45^ contain several items that are also relevant for the transparent reporting of prediction model studies using datasets from electronic healthcare records, multicentre cohort studies, or IPD-MA studies.

The executive group subsequently convened 11 times in person over four years to discuss the development and structure of TRIPOD-Cluster, the rationale for including new items, topics requiring further consideration, and the publication strategy. The initial face-to-face meetings were also used to initiate a large Delphi survey and to test the preceding versions of this TRIPOD-Cluster checklist.

A first draft of the TRIPOD-Cluster checklist was then prepared by the executive group. This draft formed the basis of an electronic questionnaire distributed to 77 Delphi participants, involving statisticians, epidemiologists, physicians, and journal editors with experience in prediction model research. The Delphi survey was opened in January 2019 and remained open for 50 days. A total of 27 modifications or new items to the original TRIPOD checklist were proposed to the Delphi participants, who rated each as “agree,” “no opinion,” or “disagree (please comment).” Three items had major disagreements (>30% scored “disagree”), and another three items had moderate disagreements (15-30% scored “disagree”), most of which were related to the (re)phrasing of newly proposed or existing items (appendix). Subsequently, a revised revision of the TRIPOD-Cluster checklist was prepared, and a second Delphi round among the same 77 participants was implemented in March 2019. All the attendant suggestions and results were then incorporated into the TRIPOD-Cluster checklist, which was discussed among participants of the annual conference of the International Society for Clinical Biostatistics (Leuven, 2019) to further refine the checklist.

## How to use the TRIPOD-Cluster checklist and the main changes from the original TRIPOD checklist

The TRIPOD-Cluster checklist is not intended to be prescriptive about how prediction model studies using clustered data should be conducted or interpreted, although we believe that the detailed guidance for each item in the TRIPOD statement will help researchers and readers for this purpose. Although TRIPOD-Cluster is to be used as a standalone reporting guideline, we also recommend using TRIPOD-Cluster alongside the original TRIPOD explanation and elaboration document,^12^ and when applicable, the PROBAST (prediction model risk-of-bias assessment tool) guidance and its accompanying explanation and elaboration on the evaluation of risk bias in prediction models.^46^ ^47^ When prediction model studies do not account for clustering (eg, because cluster identifiers were not recorded or clusters were too small), we recommend that authors explicitly report this and adopt the original TRIPOD checklist.

Reporting of all relevant information might not always be feasible, for instance, owing to word count limits of specific journals or websites. In these situations, researchers might summarise the most relevant information in a table or figure, and provide additional details in the supplementary material.

A total of 19 original TRIPOD items and subitems were modified. Some subitems from the original TRIPOD checklist were merged in TRIPOD-Cluster for simplification. These merged subitems include the definition and assessment of outcomes (TRIPOD items 6a and 6b) and predictors (TRIPOD items 7a and 7b), characteristics of included data sources (TRIPOD items 4a, 4b, 5a, 5b, 13b, and 14a), and details on the model specification (TRIPOD items 15a and 15b).

Finally, 10 new subitems were included to look at identification of multiple eligible data sources or clusters (eg, in IPD-MA studies), data preparation, risk-of-bias assessment, heterogeneity in prediction model parameters, heterogeneity in prediction model performance estimates, and cluster specific and sensitivity analyses.

The resulting TRIPOD-Cluster guidance comprises 19 main items and is presented in this manuscript (table 3; separate version in the supplementary table). An explanation for each item and subitem is described in the accompanying explanation and elaboration document, where each subitem is illustrated with examples of good reporting from published electronic healthcare records (or multicentre) and IPD-MA studies, including those listed in table 1 and table 2.

**Table 3:** TRIPOD-Cluster checklist of items to include when reporting a study developing or validating a multivariable prediction model using clustered data

#	Description	Page #
**Title and abstract**		
1	Identify the study as developing and/or validating a multivariable prediction model, the target population, and the outcome to be predicted.	
2	Provide a summary of research objectives, setting, participants, data source, sample size, predictors, outcome, statistical analysis, results, and conclusions.^*^	
**Introduction**		
3a	Explain the medical context (including whether diagnostic or prognostic) and rationale for developing or validating the prediction model, including references to existing models, and the advantages of the study design.^*^	
3b	Specify the objectives, including whether the study describes the development or validation of the model.^*^	
**Methods**		
4a	Describe eligibility criteria for participants and datasets.^*^	
4b	Describe the origin of the data, and how the data were identified, requested, and collected.	
5	Explain how the sample size was arrived at.^*^	
6a	Define the outcome that is predicted by the model, including how and when assessed.^*^	
6b	Define all predictors used in developing or validating the model, including how and when measured.^*^	
7a	Describe how the data were prepared for analysis, including any cleaning, harmonisation, linkage, and quality checks.	
7b	Describe the method for assessing risk of bias and applicability in the individual clusters (eg, using PROBAST).	
7c	For validation, identify any differences in definition and measurement from the development data (eg, setting, eligibility criteria, outcome, predictors).^*^	
7d	Describe how missing data were handled.^*^	
8a	Describe how predictors were handled in the analyses.	
8b	Specify the type of model, all model-building procedures (eg, any predictor selection and penalisation), and method for validation.^*^	
8c	Describe how any heterogeneity across clusters (eg, studies or settings) in model parameter values was handled.	
8d	For validation, describe how the predictions were calculated.	
8e	Specify all measures used to assess model performance (eg, calibration, discrimination, and decision curve analysis) and, if relevant, to compare multiple models.	
8f	Describe how any heterogeneity across clusters (eg, studies or settings) in model performance was handled and quantified.	
8g	Describe any model updating (eg, recalibration) arising from the validation, either overall or for particular populations or settings.^*^	
9	Describe any planned subgroup or sensitivity analysis, (eg, assessing performance according to sources of bias, participant characteristics, setting).	
**Results**		
10a	Describe the number of clusters and participants from data identified through to data analysed. A flow chart may be helpful.^*^	
10b	Report the characteristics overall and where applicable for each data source or setting, including the key dates, predictors, treatments received, sample size, number of outcome events, follow-up time, and amount of missing data.^*^	
10c	For validation, show a comparison with the development data of the distribution of important variables (demographics, predictors, and outcome).	
11	Report the results of the risk of bias assessment in the individual clusters.	
12a	Report the results of any across-cluster heterogeneity assessments that led to subsequent actions during the model’s development (eg, inclusion or exclusion of particular predictors or clusters).	
12b	Present the final prediction model (ie, all regression coefficients, and model intercept or baseline estimate of the outcome at a given time point) and explain how to use it for predictions in new individuals.^*^	
13a	Report performance measures (with uncertainty intervals) for the prediction model, overall and for each cluster.	
13b	Report results of any heterogeneity across clusters in model performance.	
14	Report the results from any model updating (including the updated model equation and subsequent performance), overall and for each cluster.^*^	
15	Report results from any subgroup or sensitivity analysis.	
**Discussion**		
16a	Give an overall interpretation of the main results, including heterogeneity across clusters in model performance, in the context of the objectives and previous studies.^*^	
16b	For validation, discuss the results with reference to the model performance in the development data, and in any previous validations.	
16c	Discuss the strengths of the study and any limitations (eg, missing or incomplete data, non-representativeness, data harmonisation problems).^*^	
17	Discuss the potential use of the model and implications for future research, with specific view to generalisability and applicability of the model across different settings or (sub)populations.^*^	
**Other information**		
18	Provide information about the availability of supplementary resources (eg, study protocol, analysis code, datasets).^*^	
19	Give the source of funding and the role of the funders for the present study.	

A separate version of this checklist is available in the supplementary table.

PROBAST=prediction model risk-of-bias assessment tool.

* Item text is an adaptation of one or more existing items from the original TRIPOD (transparent reporting of a multivariable prediction model for individual prognosis or diagnosis) checklist.

## Concluding remarks

TRIPOD-Cluster provides comprehensive consensus based guidance for the reporting of studies describing the development, validation, or updating of a multivariable diagnostic or prognostic prediction model using clustered data, such as those using electronic healthcare records or IPD-MA datasets (table 1 and table 2). TRIPOD-Cluster is not intended for reporting of studies in which the clustering is determined by repeated measurements of the same individuals. The checklist provides explicit guidance on the minimum required information to report, to help readers and potential model users understand how the study was designed, conducted, analysed, and inferred. We do not propose a standardised structure of reporting; rather, all the information requested in the checklist should be reported somewhere in the report or article, including supplementary material. We encourage authors to complete the checklist indicating the page number where the relevant information is reported and to submit this checklist with the article. The TRIPOD-Cluster checklist can be downloaded online (www.tripod-statement.org). Announcements relating to TRIPOD-Cluster will be made via www.tripod-statement.org and by the TRIPOD Twitter account (@TRIPODstatement). The EQUATOR Network will help disseminate and promote TRIPOD-Cluster.

## Web extra

Extra material supplied by authors

Web appendix: Supplementary information

Supplementary table: TRIPOD-Cluster checklist

## Notes

Contributors: TPAD is the guarantor. TPAD, GSC, RDR, Doug Altman, JBR, and KGMM conceived the TRIPOD-Cluster statement and developed an initial checklist. TPAD organised two Delphi rounds to obtain feedback on individual items of the TRIPOD-Cluster checklist. TPAD and KGMM prepared the first draft of the manuscript. All authors reviewed subsequent drafts and assisted in revising the manuscript. The corresponding author attests that all listed authors meet authorship criteria and that no others meeting the criteria have been omitted.

Provenance and peer review: Not commissioned; externally peer reviewed.
