# Supplementary material for: Transparent reporting of multivariable prediction models developed or validated using clustered data: TRIPOD-Cluster checklist
Source: BMJ. 2023 Feb 7;380:e071018. doi: 10.1136/bmj-2022-071018 (PMC9903175; doi:10.1136/bmj-2022-071018)
Supplement: Supplementary file 1 — Web appendix: Supplementary information [file debt071018.ww.pdf]

Appendix to TRIPOD-Cluster

Results from the Delphi surveys

Below, we present the results from both Delphi surveys. For each item, we asked whether survey participants agree with the proposed text.

Item 1

This item is based on TRIPOD item 1, which states: *"Identify the study as developing and/or validating a multivariable prediction model, the target population, and the outcome to be predicted"*.

Proposed checklist item (Delphi #1): Identify the study as developing and/or validating a multivariable prediction model, the target population, the outcome to be predicted, and the source of the data.

Actions: We revised the item text to address the main concern (i.e., "the source of the data"), and therefore did not include it in the second Delphi round.

Final Checklist item: Identify the study as developing and/or validating a multivariable prediction model, the target population, and the outcome to be predicted.

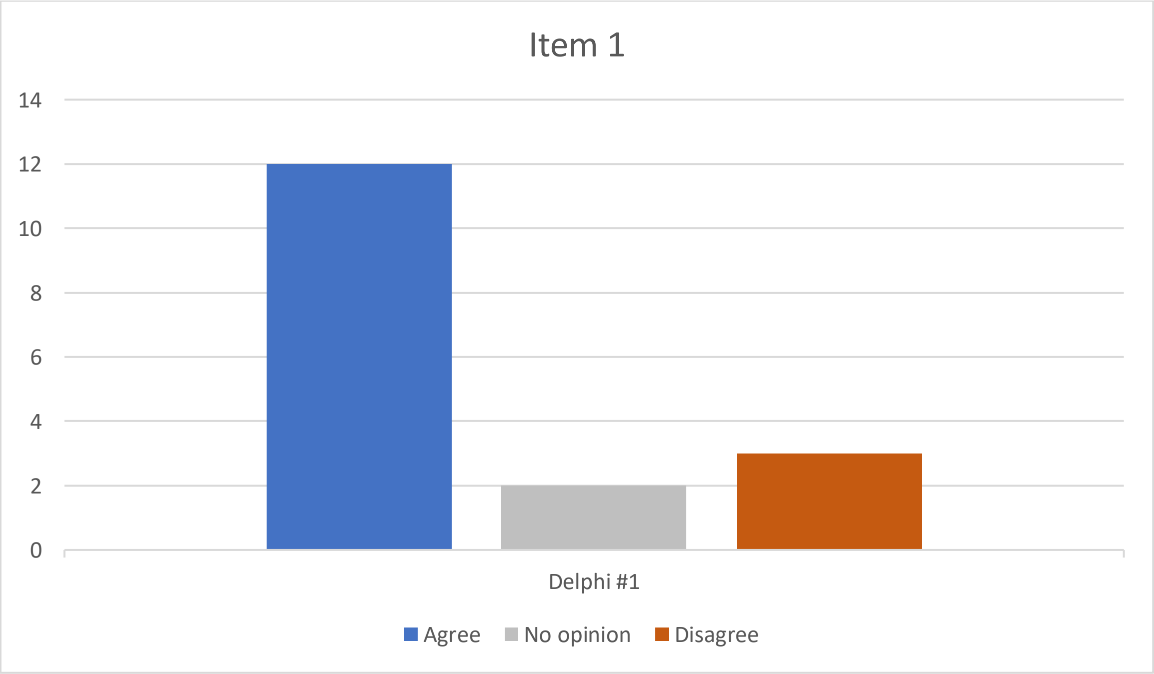

Figure 1 Results of the first Delphi round for Item 1 ("Identify the study as developing and/or validating a multivariable prediction model, the target population, and the outcome to be predicted")

Comments:

- I partially disagree as including the source of the data could make the title too long if multiple sources of data are used, e.g. linked data or different data sources for development and validation
- I agree that data source should be mentioned in the abstract but may be difficult to include in the title if IPD comes from many sources unless you mean to say something like mentioning that it is an IPD-MA.

- Is this for the title or title/abstract? I agree that some indication of the source or at least type of data should be given in the title.
- The change is ok, but the source is also noted in item #2. One comment is that the phrase "multivariable prediction model" is now too prescriptive with all the new machine learning prediction models. I would just change this to "prediction model", and have people say what type of prediction model (there are so many options now).
- Not convinced the source of data is essential in the title but it is essential in the abstract. Even more so since the source of the data may have a long name.
- The source is important, but I don't feel it's essential for the title.

Item 2

This item is based on TRIPOD item 2, which states: *"Provide a summary of objectives, study design, setting, participants, sample size, predictors, outcome, statistical analysis, results, and conclusions"*.

Proposed checklist item (Delphi #1): Provide a summary of research objectives, setting, participants, data source, sample size, predictors, outcome, statistical analysis, results, and conclusions.

Actions: We did not make any further revisions to the item text. This item was not assessed in the second Delphi round because there were no disagreements.

Final Checklist item: Provide a summary of research objectives, setting, participants, data source, sample size, predictors, outcome, statistical analysis, results, and conclusions.

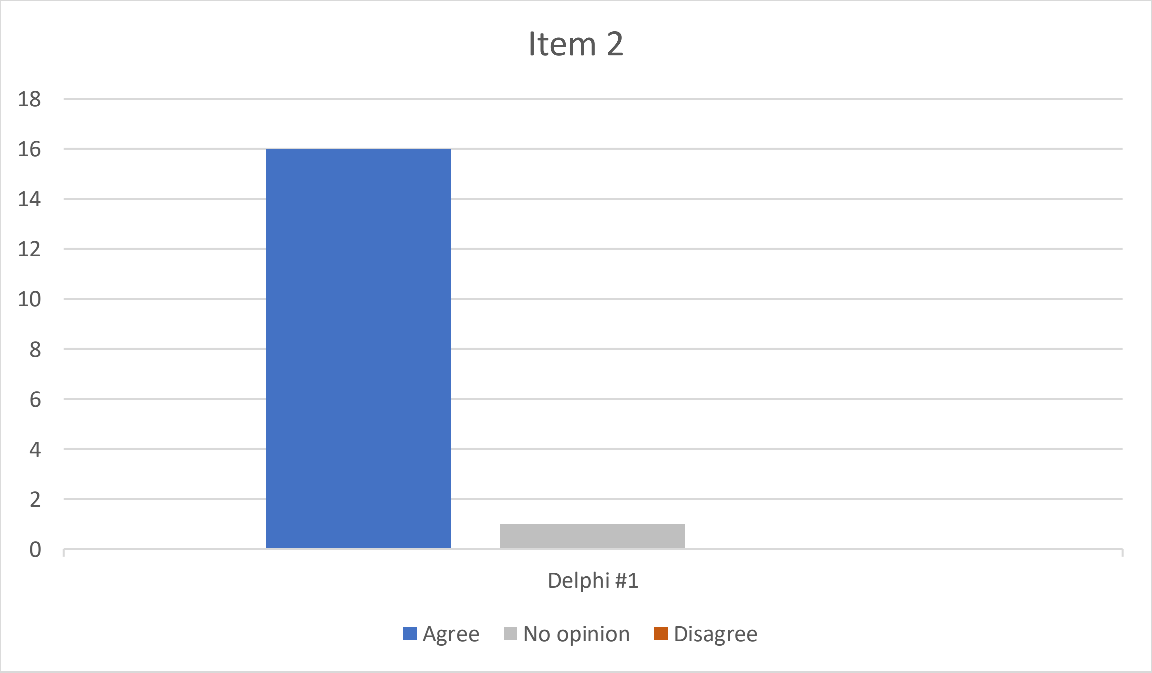

Figure 2 Results of the first Delphi round for Item 2 ("Provide a summary of research objectives, setting, participants, data source, sample size, predictors, outcome, statistical analysis, results, and conclusions.")

Comments:

- Data source quite general perhaps. One may say 'EHR', but not whether it is from one or several sites.
- Sample size may differ for different parts of the analysis so could be difficult to report in the abstract e.g. if validating more than one model or validating several models using different subsets of the data. In guidance alongside the checklist, might want to note that the sample size should be that used for each analysis rather than total IPD available as we cannot always use it all.

Item 3a

This item is based on TRIPOD item 3a, which states: *"Explain the medical context (including whether diagnostic or prognostic) and rationale for developing or validating the multivariable prediction model, including references to existing models"*.

Proposed checklist item (Delphi #1): Explain the medical context (including whether diagnostic or prognostic) and rationale for developing or validating the multivariable prediction model, including references to existing models, and the advantages of the source of data (e.g. size, representativeness).

Actions: We revised the text that was raising concerns (*"[…] , and the advantages of the source of data (e.g. size, representativeness)."*), and did not assess the revised text in the second Delphi round.

Final Checklist item: Explain the medical context (including whether diagnostic or prognostic) and rationale for developing or validating the prediction model, including references to existing models, and the advantages of the study design.

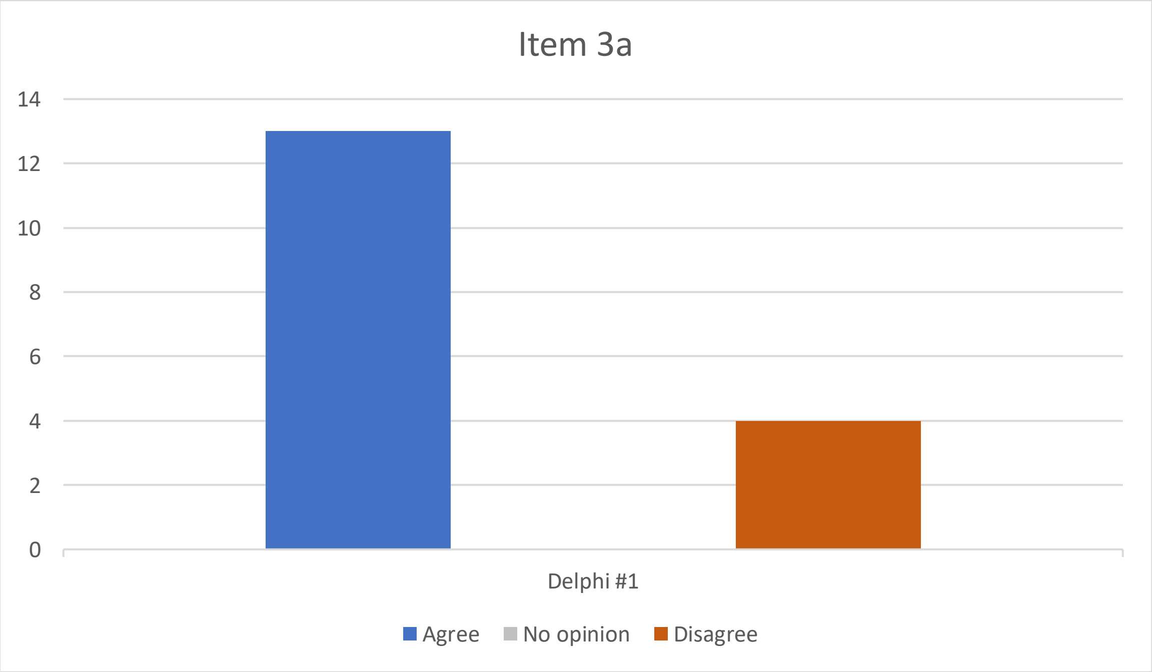

Figure 3 Results of the first Delphi round for Item 3a ("Explain the medical context (including whether diagnostic or prognostic) and rationale for developing or validating the prediction model, including references to existing models, and the advantages of the study design.")

Comments:

- Advantage can also relate to how/when a prediction is needed in real life, I guess this is broadly covered under 'representativeness'. Heterogeneity may also be a good advantage?
- A phrasing issue: maybe add 'particular' into the phrase 'the source of data', to avoid that authors simply state that big data has advantages in general
- Can't have "source data" everywhere. Pick one place item #1, #2, #3 and leave it there.
- Rather than "advantages" perhaps "properties" may be a better term to allow for more generic information about the data source e.g. time span, how the data were collated, etc. Asking researchers to focus on advantages at this point may lead to duplication later in the discussion when strengths and limitations are discussed.

- I think this is more relevant to the discussion. Details of data source validation, for example, can be included in Methods. But I don't see why this is necessary for the introduction
- Use of big data to construct a model may be out of convenience or opportunistic, e.g. "making use of" routinely collected data. Not necessarily an advantage.

Item 3b

This item is a minor adaptation from TRIPOD item 3b ("*Specify the objectives, including whether the study describes the development or validation of the model, or both.*") and was therefore not assessed in any Delphi rounds.

Final Checklist item: Specify the objectives, including whether the study describes the development or validation of the model.

Item 4a

This item is based on TRIPOD item 5b, which states: "*Describe eligibility criteria for participants*".

Proposed checklist item (Delphi #1): Describe eligibility criteria for participants and data sources.

Actions: We rephrased “data sources” by “datasets” to harmonize terminology adopted by TRIPOD-Cluster. This item was not assessed in the second Delphi round because most participants agreed on its phrasing.

Final Checklist item: Describe eligibility criteria for participants and datasets.

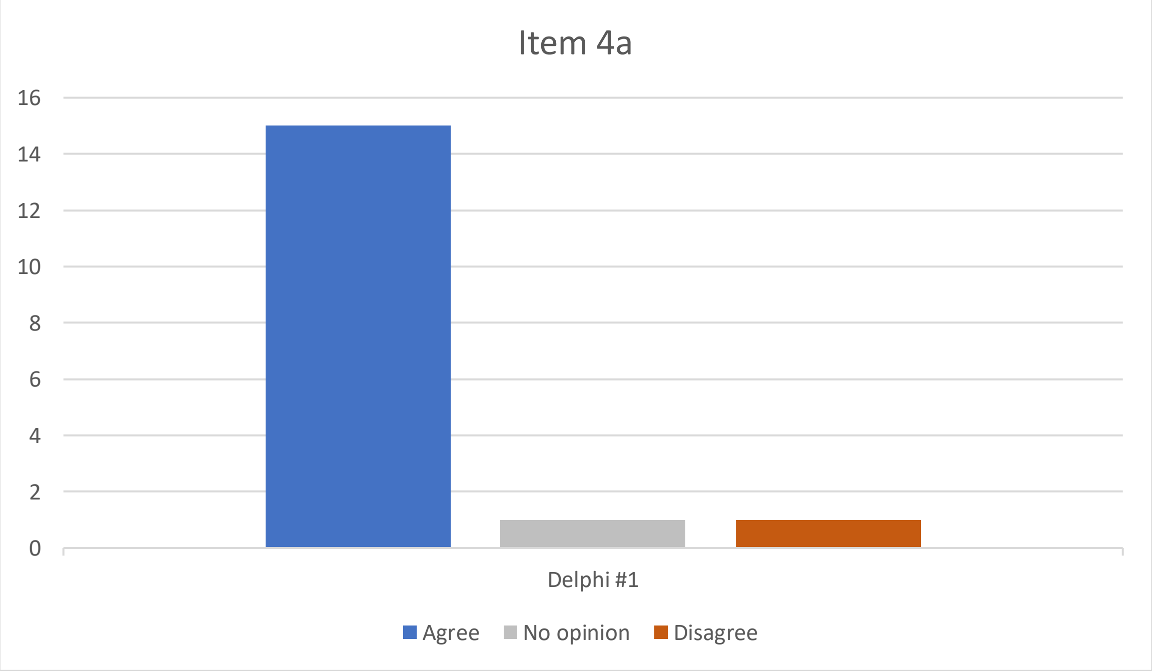

Figure 4 Results of the first Delphi round for Item 4a (“Describe eligibility criteria for participants and datasets.”)

Comments:

- Although data sources may be 'eligible', there may be less variability in patient characteristics from some sources than others, for example if that particular study/dataset had stricter inclusion/exclusion criteria. Wouldn't be addressed by this item, but should be summarised somewhere.
- I agree that eligibility of different sources should be described if the data consists of IPD data or of a combination of different sources. However, if one makes use of a large registry (eg CPRD), which is also considered big data, eligibility of data sources is not relevant.
- I like less wordy.
- Agree but not sure I fully understand the bit about data sources. Do you mean eligibility criteria for selecting data sources?

Item 4b

This is a new item that was not included in the original TRIPOD checklist.

Proposed checklist item (Delphi #1): Describe how the data were identified, requested and collected.

Actions: This item was not assessed in the second Delphi round because there were no disagreements about its phrasing and relevance.

Final Checklist item: Describe the origin of the data, and how the data were identified, requested and collected.

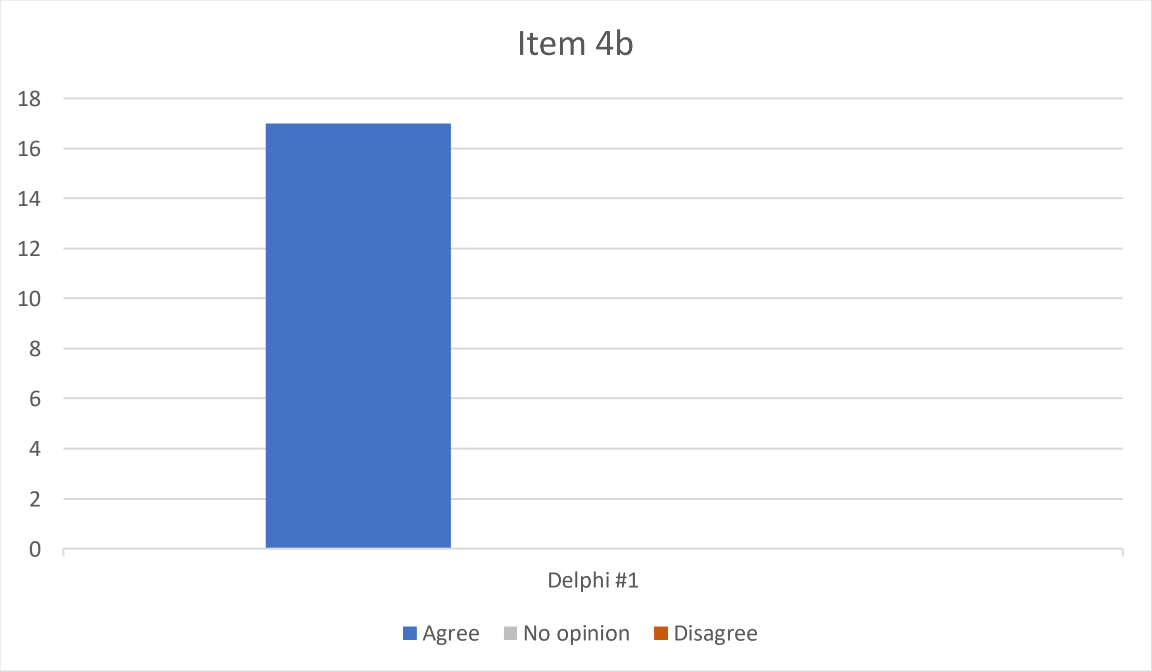

Figure 5 Results for the first round of the Delphi for Item 4b ("Describe the origin of the data, and how the data were identified, requested and collected.")

Comments:

- Plus how these were harmonised? (May follow later)
- 1) Not sure what 'data were identified' means in a non-IPD big data setting. 2) Regarding wording: 'data were identified' might be confused with patient (de)identification

Item 5

This item is a minor adaptation from TRIPOD item 8 (“*Explain how the study size was arrived at.*”) and was therefore not assessed in any of the Delphi rounds.

Final Checklist item: Explain how the sample size was arrived at.

Item 6a

This item is a minor adaptation from TRIPOD item 6a (“*Clearly define the outcome that is predicted by the prediction model, including how and when assessed.*”) and was therefore not assessed in any of the Delphi rounds.

Final Checklist item: Define the outcome that is predicted by the model, including how and when assessed.

Item 6b

This item is a minor adaptation from TRIPOD item 7a (“*Clearly define all predictors used in developing the multivariable prediction model, including how and when they were measured*.”) and was therefore not assessed in any of the Delphi rounds.

Final Checklist item: Define all predictors used in developing or validating the model, including how and when measured.

Item 7a

This is a new item that was not included in the original TRIPOD checklist.

Proposed checklist item (Delphi #1): Describe how data were cleaned including any harmonization and linkage.

Proposed checklist item (Delphi #2): Describe how the data were prepared for analysis, including any cleaning, harmonization and linkage.

Final Checklist item: Describe how the data were prepared for analysis, including any cleaning, harmonisation, linkage, and quality checks.

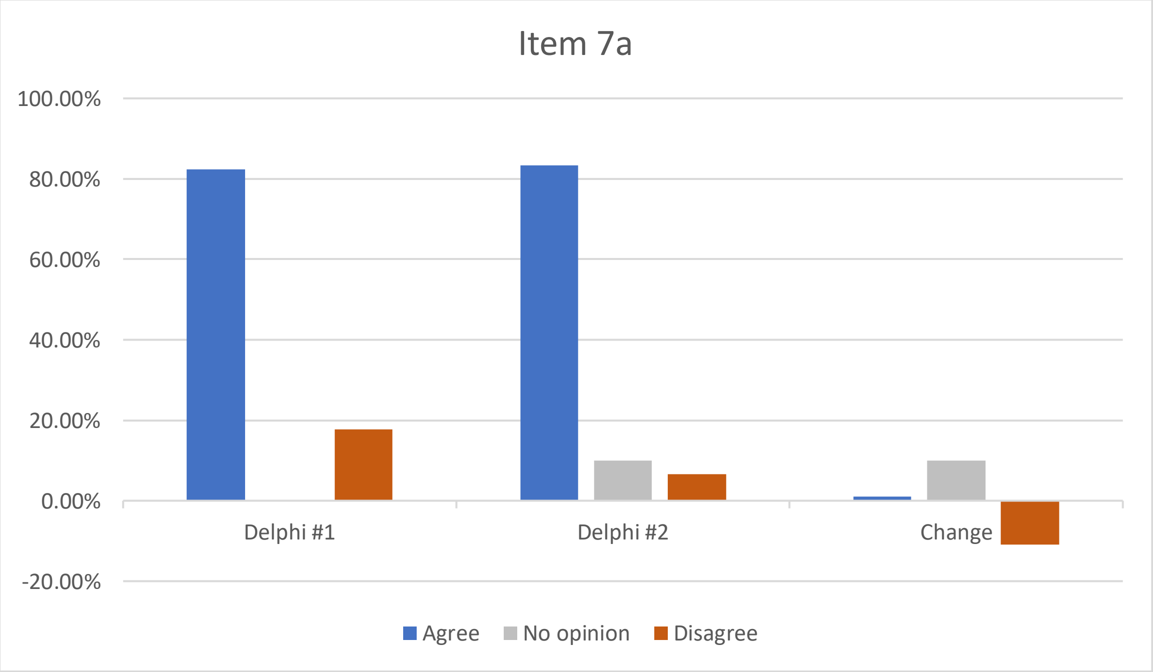

Figure 6 Results of the first Delphi round (N=17), the second Delphi round (N=30), and changes in agreement between the two rounds of the Delphi for Item 7a ("Describe how the data were prepared for analysis, including any cleaning, harmonisation, linkage, and quality checks.")

Comments Delphi #1:

- Partially disagree. It depends on the amount of information required on data cleaning as this is often extensive and journals are unlikely to want this in detail
- Should have been part of standard TRIPOD? '80% of time is spent in cleaning and preparation of data'?
- Yes!
- 'Describe how data were harmonized and linked'? 'Describe how data were cleaned' sounds quite large to me.
- The phrasing is rather vague, which makes it difficult to understand how much detail is required here, and what is meant by harmonization and linkage. I suspect that in IPD research the terminology is more common than in other big data areas. (Or at least it may have a different interpretation). Also, this is not necessary in big data which does not need to be combined (eg CPRD)
- I like it.
- including how predictors were constructed (relevant for EHR records that may require data manipulation)

Comments Delphi #2:

- Should not necessarily be in main study report. Have a protocol and SAP as supplementary material.
- I do not think that journals offer enough extra space in the manuscript for this information, so many times this item will be empty.
- I agree with the proviso that a concise description can be given in the Methods section of a paper, with more detail if needed in a supplement. There is potential for this description to be very long which would be a problem in journals with a tight word limit.

Item 7b

This is a new item that was not included in the original TRIPOD checklist.

Proposed checklist item (Delphi #1): Describe how risk of bias was assessed (e.g. using PROBAST) for each study or setting, addressing participant selection, predictors and outcome.

Proposed checklist item (Delphi #2): Describe how potential sources of bias were assessed (e.g. using PROBAST).

Final Checklist item: Describe the method for assessing risk of bias and applicability in the individual clusters (e.g., using PROBAST).

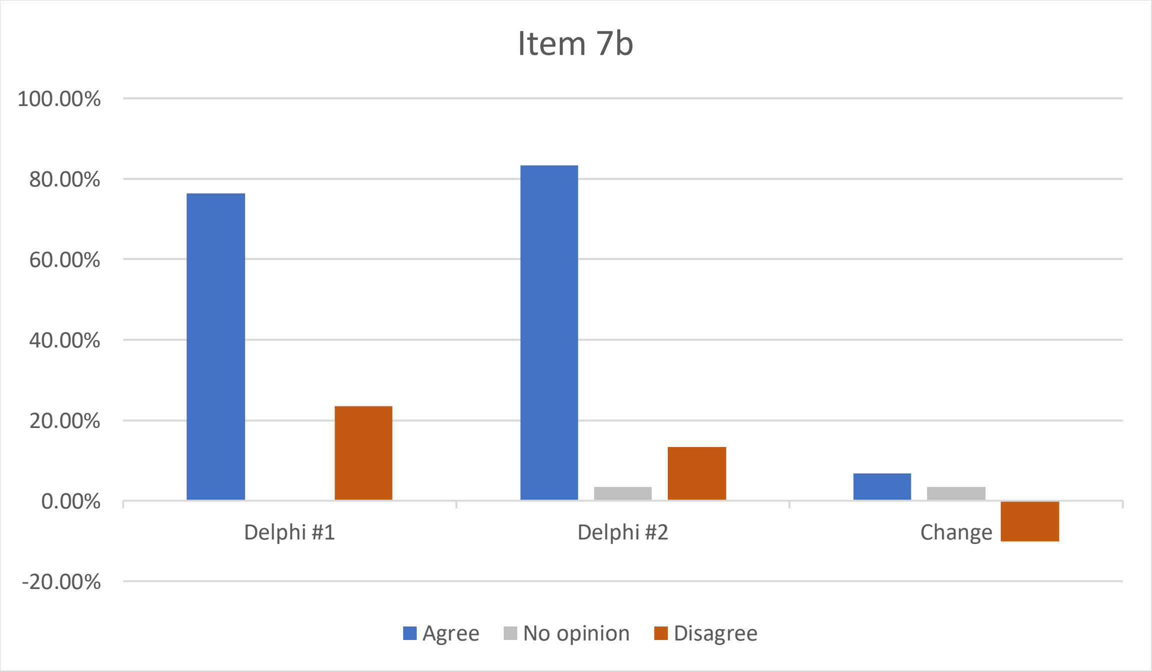

Figure 7 Results of the first Delphi round (N=17), the second Delphi round (N=30), and changes in agreement between the two rounds of the Delphi for Item 7b ("Describe the method for assessing risk of bias and applicability in the individual clusters (e.g. using PROBAST).")

Comments Delphi #1:

- I think risk of bias is better evaluated by independent researchers - or have I misunderstood something here.
- What's the alternative for PROBAST?
- Not completely sure. Could this lead to excluding databases/studies?
- Quality and biases associated with the data considered here too or elsewhere? e.g. availability bias with IPD?
- Separate to this but perhaps PROBAST in this setting needs to consider the studies in relation to some 'target' e.g. participants. Questions consistency within a study/dataset but what about across different datasets when they will be combined for IPD-MA? As mentioned before, the case-mix may be narrower in some studies than others but this will not contribute towards RoB as far as I understand. Also, outcome may be defined/measured slightly differently in different data sources.
- This is mainly relevant for IPD context; in context of combining different datasets the individual items are important as well, but not using the wording that is common in the IPD-

field. Also PROBAST may not be known in this area. For big data concerning large registries, the wording "for each" is not relevant and might be confusing.

- Too wordy. Why now just say "Identify potential sources of bias"?

Comments Delphi #2:

- For a registry-type study, this item may be confusing. The background document may give some explicit guidance. We may also produce two 1-pager alongside the checklist explaining nonintuitive issues from the perspective of an IPD-meta-analysis type of study and from a registry-type study.
- Consider the wording 'quality of the data' as I am not sure if the phrasing bias would apply to studies with electronic health records.
- PROBAST is only one of the options; quite a complex instrument
- This is more relevant for independent reviews of the publication - e.g. systematic reviews. Authors are likely to be biased in assessing sources of bias in their own publications.
- As TRIPOD relates to reporting of study that is conducted by the investigators and the investigators themselves will follow TRIPOD, should this item be rephrased as "Describe how potential sources of bias were addressed (e.g. those listed in PROBAST)".

Item 7c

This is a new item that was not included in the original TRIPOD checklist.

Proposed checklist item (Delphi #1): Indicate which data were used for development and which for validation, and how they were chosen.

Actions: Based on the feedback, we decided that a new item is not required for this reporting issue and adopted the text of TRIPOD item 12 (*"For validation, identify any differences from the development data in setting, eligibility criteria, outcome, and predictors."*).

Proposed checklist item (Delphi #2): For validation, identify any differences in definition and measurement from the development data (e.g. setting, eligibility criteria, outcome, predictors).

Final Checklist item: For validation, identify any differences in definition and measurement from the development data (e.g. setting, eligibility criteria, outcome, predictors).

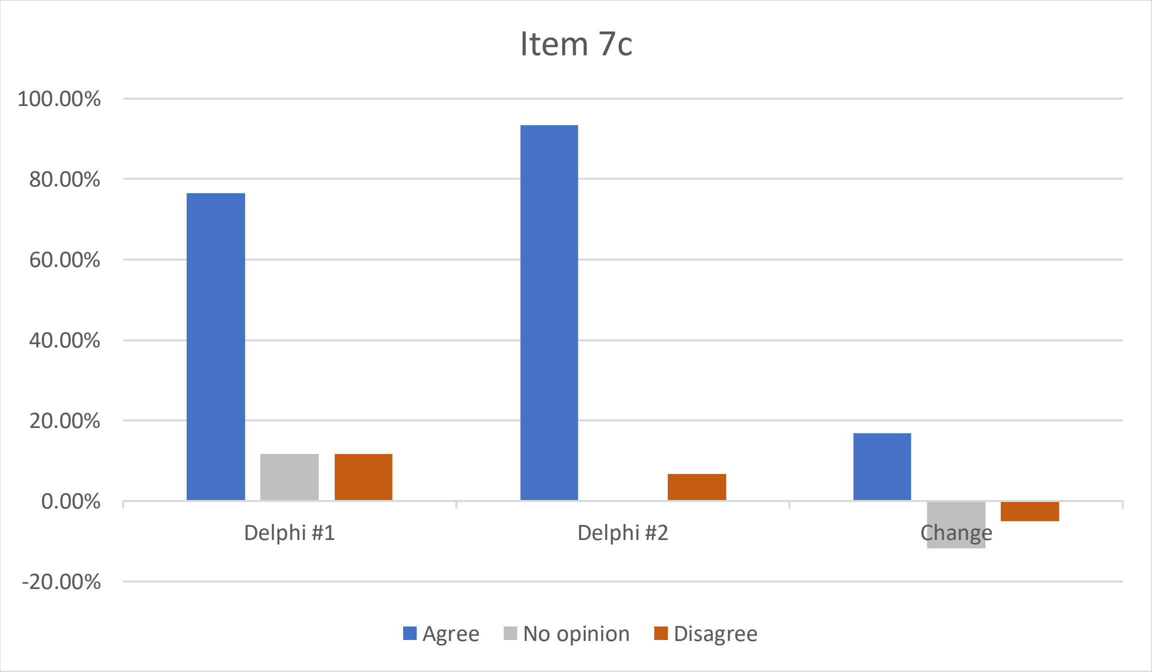

Figure 8 Results of the first Delphi round (N=17), the second Delphi round (N=30), and changes in agreement between the two rounds of the Delphi for Item 7c ("For validation, identify any differences in definition and measurement from the development data (e.g., setting, eligibility criteria, outcome, predictors).")

Comments Delphi #1:

- This suggests a data splitting procedure, as if we need to split in development vs validation. Internal validation procedures are recommended, and internal-external validation. Not keeping validation data separate.
- I would formulate this more clearly, this may suggest a train-test split but that is surely not what it means to say. Does this specifically focus on external validation?
- Does this cover a wider array of 'how validation was done'? Question feels like it implies external validation, but would this item also cover describing internal validation (under development)?
- Strongly agree with the 'how they were chosen'.
- Why is this different from item 4a of the regular TRIPOD statement?

- This encourages "hold-out" samples, which are less efficient. Also could be confusing with k-fold validation etc. Why say "Describe the internal and external cross-validation procedures".
- Explicitly mention cross-validation or internal-external validation?

Comments Delphi #2:

- Are differences in definition/measurement between clusters dealt with anywhere?
- I strongly agree. This is a commonly under-reported information I would be happy to see more often in validation studies.
- Pretty vague
- There may also be differences in measurement (e.g. different assay or machine for biomarkers) across clusters.
- Language implies that only external validation is entertained.
- Would the suggestion: "Preferably identify possible differences with a clinical expert" or something like that be helpful?

Item 8a

Proposed checklist item (Delphi #2): Describe how predictors were handled in model development.

Final Checklist item: Describe how predictors were handled in the analyses.

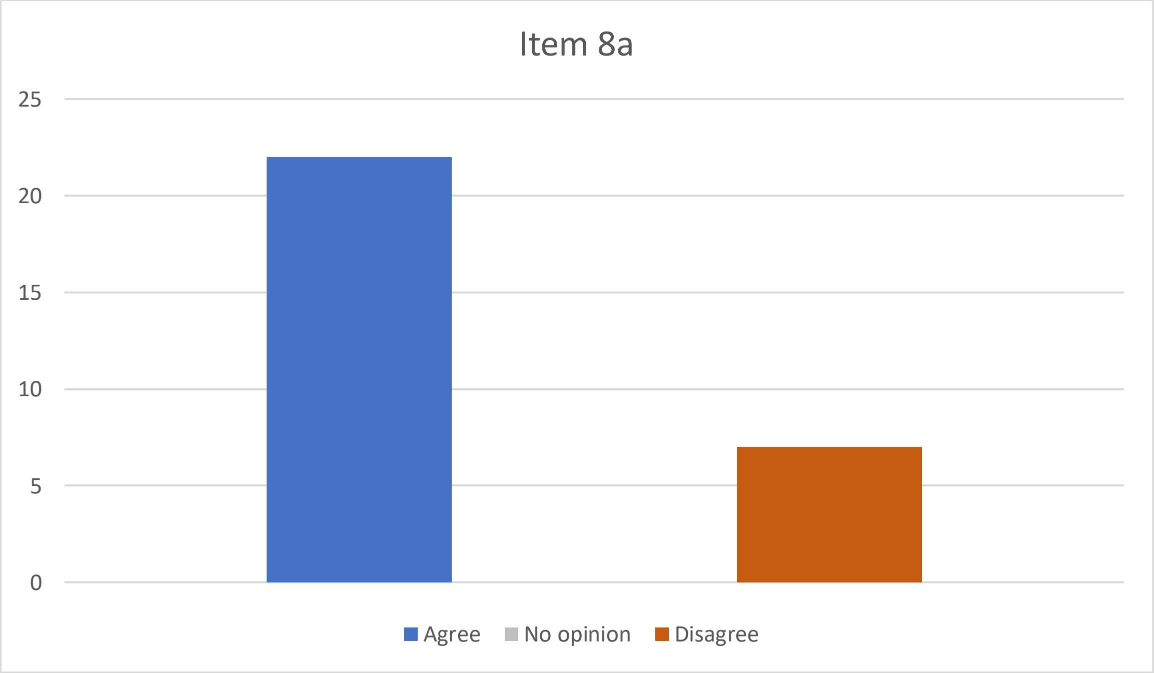

Figure 9 Results of the second Delphi round for Item 8a ("Describe how predictors were handled in the analyses.")

Comments:

- But perhaps a bit vague here as it stands Do you specifically refer to continuous predictors?
- Could this be made more specific?
- Perhaps even a bit stronger Describe all relevant details how …
- I think this item is too broad, does model development also include decisions about predictor coding, linearity checking. That is not clear.
- I think it also needs an example as you have for the other items. I presume it refers to splines/FPs for continuous variables etc.?
- A more precise sentence with specific points could improve the reporting of this very important step.
- I think "handled" is vague. I'd put at least some (e.g., XXX) in there.
- Is it clear from the section of TRIPOD where this item is listed that this relates to model development? Just to make sure this item isn't listed at "validation". That said, the formulation of the item in itself is rather vague. What is meant by "were handled"? Can this be specified in more detail? For example by adding a "e.g. blablabla".
- 'how predictors were handled' is very unspecific. I suggest to add an example what is meant here (e.g., do you mean that it should be described if predictors were dichotomized, transformed (based on which grounds), ...?)

Item 8b

This item is based on TRIPOD item 10b, which states: "*Specify type of model, all model-building procedures (including any predictor selection), and method for internal validation*".

Proposed checklist item (Delphi #1): Specify type of model, all model-building procedures (e.g. any predictor selection and penalization), and method for internal validation.

Proposed checklist item (Delphi #2): Specify the type of model, all model-building procedures (e.g. any predictor selection and penalization), and method for validation.

Final Checklist item: Specify the type of model, all model-building procedures (e.g. any predictor selection and penalization), and method for validation.

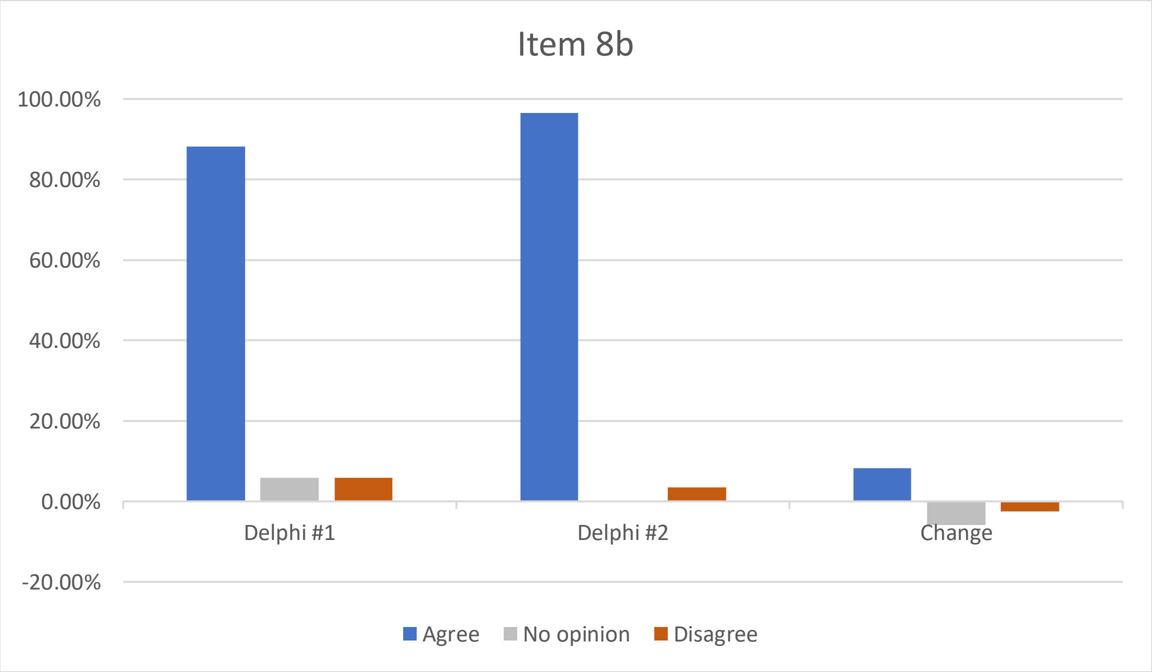

Figure 10 Results for the first Delphi round (N=17), the second Delphi round (N=29), and changes in agreement between the two rounds of the Delphi for item 8b ("Specify the type of model, all model-building procedures (e.g., any predictor selection and penalization), and method for validation.")

Comments Delphi #1:

- I would specifically include 'handling of continuous predictors'
- Suggest you call it "model or algorithm". For neural networks they should specify the network architecture. Include tuning parameters in examples. Note that sometimes a small internal validation set is used to select the final model or tune parameters. You need to include language used in the machine learning literature, though the principles are the same.

Comments Delphi #2:

- Closely linked to 8c
- categorisation is still very popular. would it make sense asking for details here (e.g. ...details about categorisation) or is that done in another item?

Item 8c

This is a new item that was not addressed by the original TRIPOD checklist.

Proposed checklist item (Delphi #1): Describe how any heterogeneity in case-mix distributions and model parameters (e.g. intercept, baseline survival, predictor effects) was handled.

Proposed checklist item (Delphi #2): Describe how any heterogeneity (e.g., across data sources or settings) in model parameters was handled during model development.

Final Checklist item: Describe how any heterogeneity across clusters (e.g., studies or settings) in model parameter values was handled.

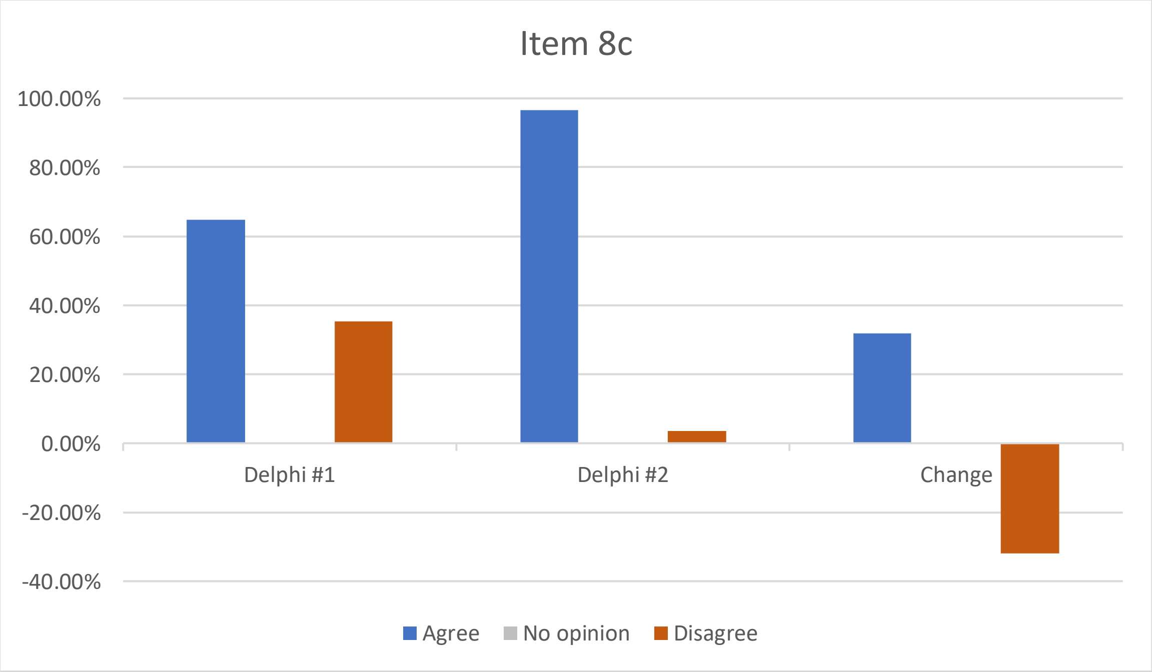

Figure 11 Results for the first Delphi round (N=17), the second Delphi round (N=29), and changes in agreement between the two rounds of the Delphi for item 8c ("Describe how any heterogeneity across clusters (e.g., studies or settings) in model parameter values was handled.").

Comments Delphi #1:

- Suggest to simplify to: Describe how any heterogeneity in case-mix distributions and model parameters was handled. Modern ML methods may not have an explicit intercept.
- Strongly agree with this addition, important aspect especially with clustered data (IPD/EHR with multiple centres) Maybe should be ended with '.. handled during development?' with 8f below in mind?
- My feeling is that more needs to be said about the heterogeneity across studies in other places, not just how it was handled. See comments on other items.
- Mainly relevant in IPD setting, but not necessarily in other big data approaches
- Do you mean "heterogeneity in model parameters" or just to describe model parameters? Many new models do not have parameters and may not even have "variables", such as those using images.
- This is way too vague...what type of heterogeneity is being referred to here? This will only show up in the parameter SE under certain modeling conditions etc. I don't think this is necessary.

- Heterogeneity in parameters across what? Several databases or data sources? I agree with the concept, but you need to mention this in the context of multiple data sets/sources (e.g. in an IPD meta-analysis), otherwise I don't think the Item makes sense. E.g. "If IPD from multiple data sources are used,..." Also, "heterogeneity in case-mix distribution" might not be very accessible phrasing to a broad audience.
- Strongly agree if relevant! Big data / EHR includes data from single setting.

Comments Delphi #2:

- This is also relevant for validation, but I guess this is what you refer to in 8f?
- To my opinion unclear
- I'm wondering what sort of answers would be "acceptable"? Most people just carry on and acknowledge differences between datasets etc.
- Model parameters is quite broad .. in particular, heterogeneity in performance is relevant?

Item 8d

This item was added as a separate item after the two Delphi rounds, to distinguish between the information requested for a prediction model development study (see item 12b). This item is identical to TRIPOD item 10c (“*For validation, describe how the predictions were calculated*.”).

Final Checklist item: For validation, describe how the predictions were calculated

Item 8e

This item is based on TRIPOD item 10d, which states: "*Specify all measures used to assess model performance and, if relevant, to compare multiple models*".

Proposed checklist item (Delphi #1): Specify all measures used to assess model performance (e.g. calibration and discrimination) and, if relevant, to compare multiple models.

Proposed checklist item (Delphi #2): Specify all measures used to assess model performance (e.g. calibration and discrimination) and, if relevant, to compare multiple models.

Final Checklist item: Specify all measures used to assess model performance (e.g. calibration*,* discrimination*, and* decision curve analysis) and, if relevant, to compare multiple models.

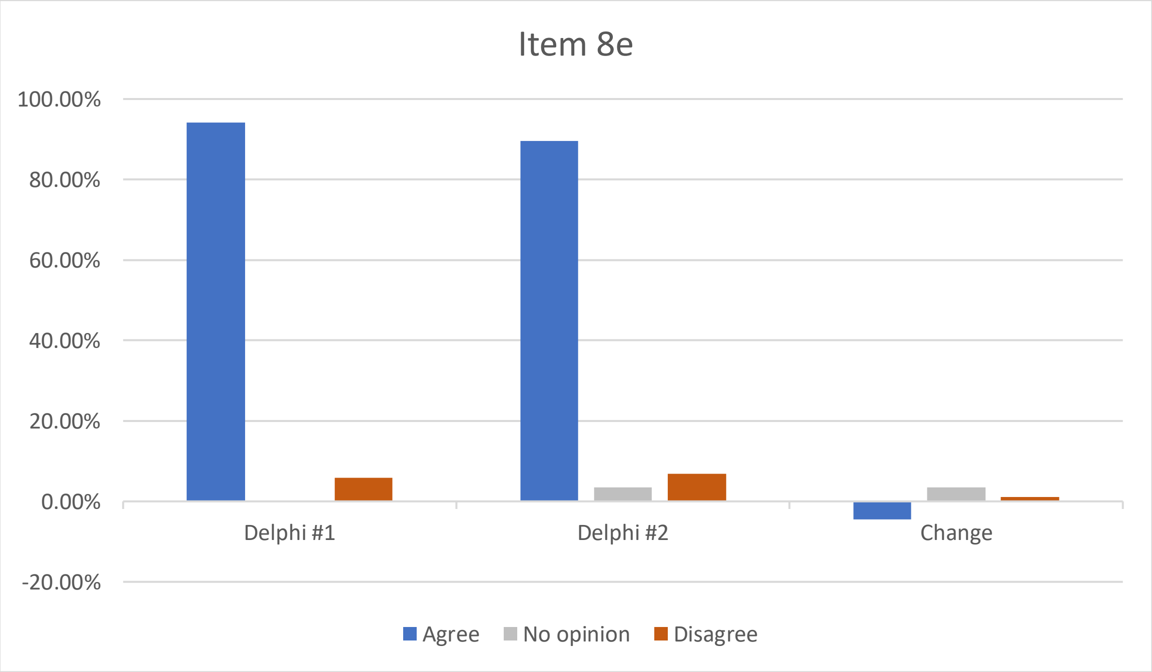

Figure 12 Results of the first Delphi round (N=17), the second Delphi round (N=29), and changes in agreement between the two rounds of the Delphi for item 8e ("Specify all measures used to assess model performance (e.g. calibration, discrimination, and decision curve analysis) and, if relevant, to compare multiple models.")

Comments Delphi #1:

- Leave as it was; R2 and decision analytic measures might be relevant to some
- Also mention assessment of heterogeneity here? (ah, that in the next item...)

Comments Delphi #2:

- Add "justify"
- Calibration and discrimination are important; but measures such as R2, or decision-analytic (NB, RU), may also be relevant. So, suggest to drop "(e.g. calibration and discrimination)"

Item 8f

This is a new item that was not addressed by the original TRIPOD checklist.

Proposed checklist item (Delphi #1): Describe how any heterogeneity in model performance was handled and quantified.

Proposed checklist item (Delphi #2): Describe how any heterogeneity (e.g., across data sources or settings) in model performance was handled and quantified.

Final Checklist item: Describe how any heterogeneity across clusters (e.g., studies or settings) in model performance was handled and quantified.

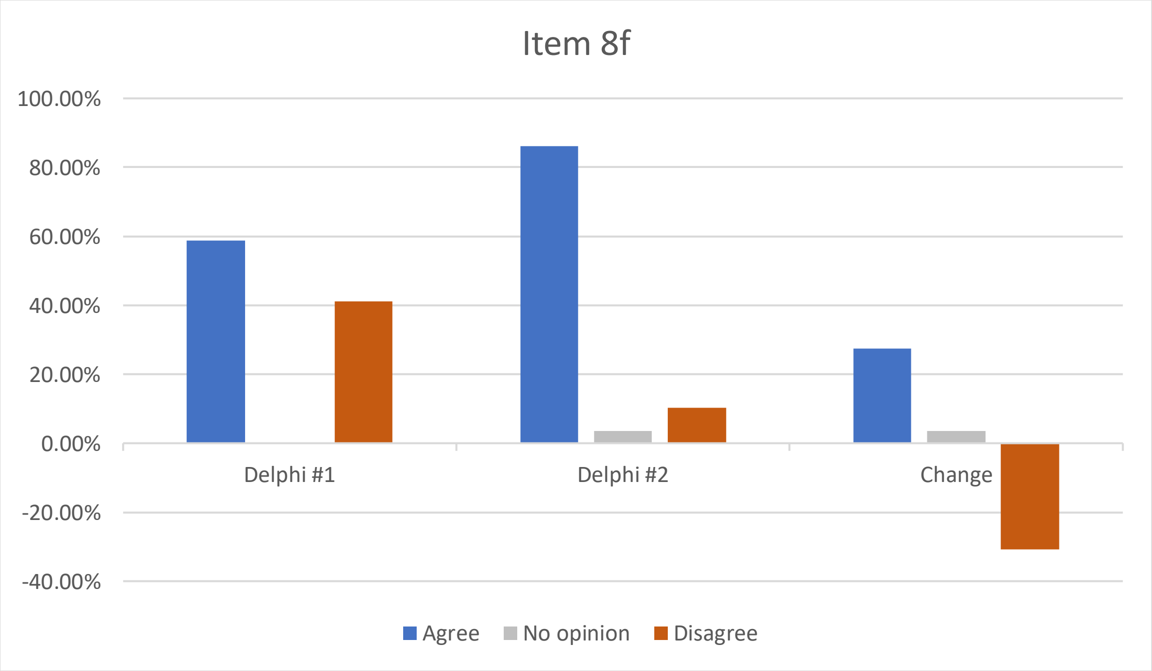

Figure 13 Results of the first Delphi round (N=17), the second Delphi round (N=29), and changes in agreement between the two rounds of the Delphi for item 8f ("Describe how any heterogeneity across clusters (e.g., studies or settings) in model performance was handled and quantified.")

Comments Delphi #1:

- This is too vague; something on "setting" ?
- It is unclear what heterogeneity this refers to.
- This to me makes the assumption that performance is assessed within clusters and pooled. Perhaps use 'Describe if and how...' as it may not be done or may not be possible e.g. if rare outcome so no/few events in some clusters.
- See my remarks at 8c, only relevant in IPD context
- Not clear. Heterogeneity over what? Different data sources? Different models?
- Just say: "Provide uncertainty estimates for any claims about model performance".
- Again, as in my comment for 8c, it needs to be clarified across where researchers should handle/quantify heterogeneity.
- Strongly agree if relevant! Big data / EHR includes data from single setting.

Comments Delphi #2:

- As above - this will be very difficult to answer in my opinion so you might need to give some
- examples.

- If applicable. Big Data may come from multiple sources, but each source could deliver new
- columns (variables) instead of new rows (observations from varying sources or settings).
- OK, perhaps 8c + 8f make for a nice set
- maybe change the order of "handled" and "quantified". I guess you'll first quantify the
- heterogeneity before you handle it (although you may make certain decision up front that may limit heterogeneity or account for heterogeneity).
- Earlier you described during question 7 'Difference between settings'. How is this exactly different? And should it not use the same terminology?

Item 8g

This item is based on TRIPOD item 10e, which states: *"Describe any model updating (e.g., recalibration) arising from the validation, if done"*.

Proposed checklist item (Delphi #1): Describe any model updating (e.g., recalibration) arising from the validation, either overall or for particular datasets or settings.

Proposed checklist item (Delphi #2): Describe any model updating (e.g., recalibration) arising from the validation, either overall or for particular populations or settings.

Final Checklist item: Describe any model updating (e.g., recalibration) arising from the validation, either overall or for particular populations or settings.

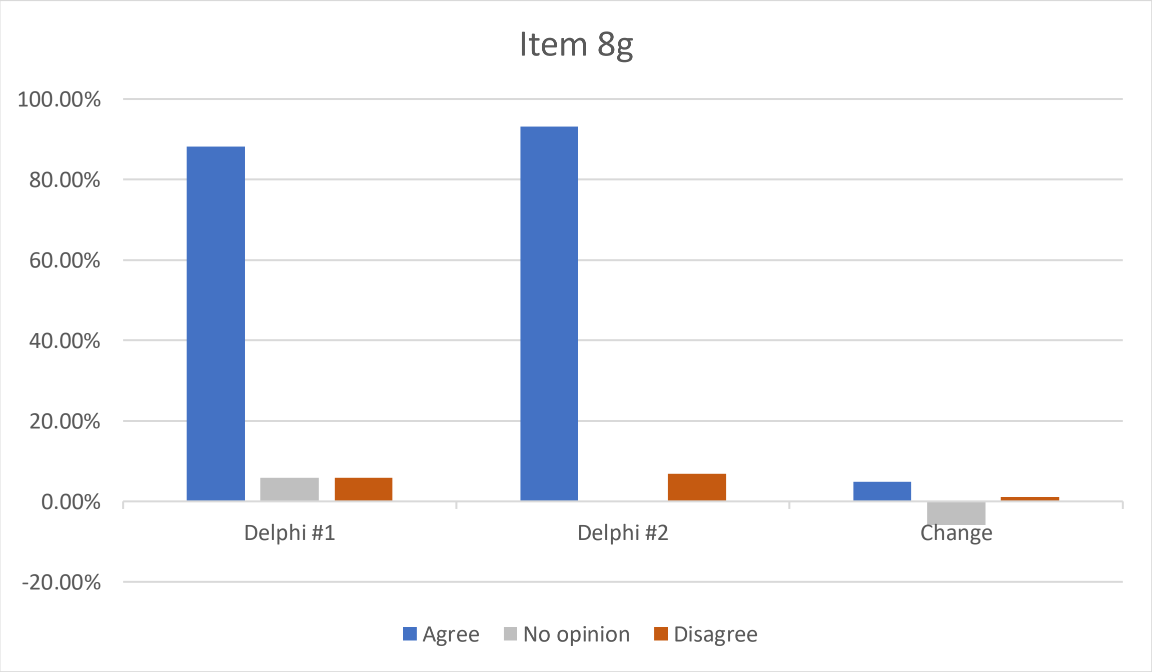

Figure 14 Results of the first Delphi round (N=17), the second Delphi round (N=29), and changes in agreement between the two rounds of the Delphi for item 8g ("Describe any model updating (e.g., recalibration) arising from the validation, either overall or for particular populations or settings.").

Comments Delphi #1

- Here "particular datasets or settings" is added; may be simplified to: "settings"; a dataset is not of interest per se; only as representing a setting
- The addition is only relevant in IPD setting. So I would leave it out here, and mention at explanation of the particular item

Comments Delphi #2:

- But validation performance 'as is' (before updating) should always be presented, right?
- Allow for more and newer creative solutions, such as seamless adaptive designs
- So long as there is the option to say updating was not required.
- And whether the updating was revalidated

Item 9

This is a new item that was not addressed by the original TRIPOD checklist.

Proposed checklist item (Delphi #1): Specify any subgroup or sensitivity analysis, e.g. assessing performance according to risk of bias, participant characteristics, setting.

Proposed checklist item (Delphi #2): Describe any planned subgroup or sensitivity analysis, e.g. assessing performance according to sources of bias, participant characteristics, setting.

Final Checklist item: Describe any planned subgroup or sensitivity analysis, e.g. assessing performance according to sources of bias, participant characteristics, setting.

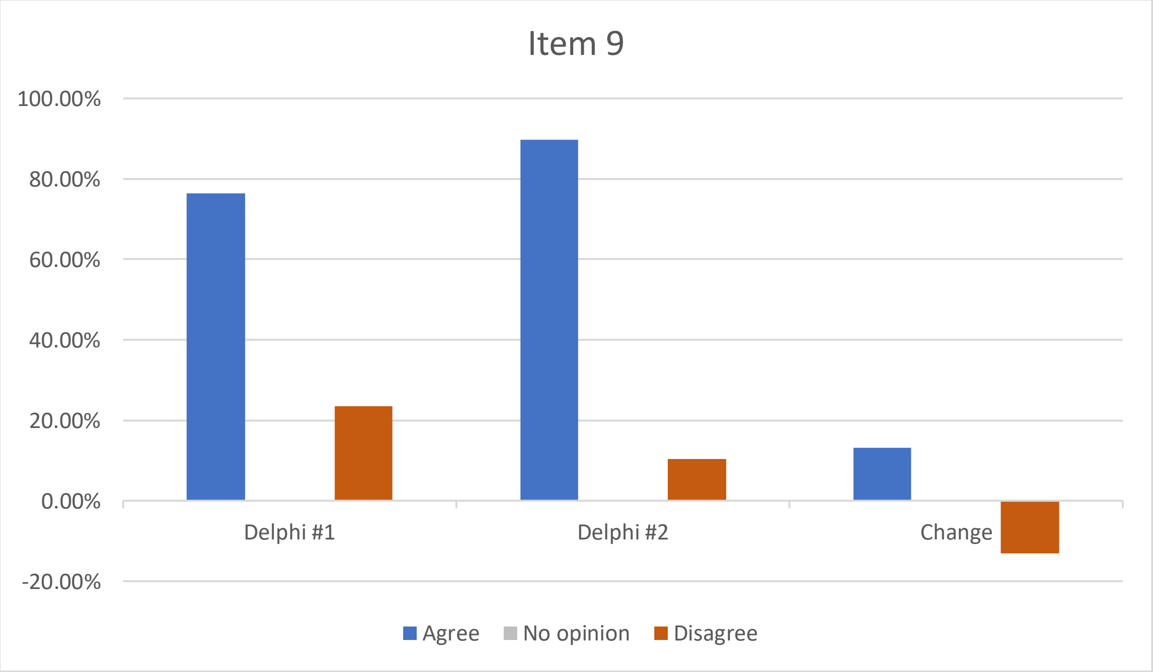

Figure 15 Results of the first Delphi round (N=17), the second Delphi round (N=29), and changes in agreement between the two rounds of the Delphi for item 9 ("Describe any planned subgroup or sensitivity analysis, e.g. assessing performance according to sources of bias, participant characteristics, setting.")

Comments Delphi #1:

- Leave this optional; including an item suggests it should be done; while modeling interactions is far more powerful and informative than subgrouping (see Harrell)
- assessing performance according to risk of bias and according to setting mainly relates to IPD data, not to large registry databases.
- This sounds like a meta-analysis. Is that your intent? I don't necessarily disagree, but would like clarification.
- Too prescriptive....
- according to risk of bias of what? This might need more clarification

Comments Delphi #2:

- One obvious source of heterogeneity are the natural clusters within a clustered data source. Do we want to highlight this?
- But posthoc analyses can also be described, if clearly labeled as posthoc?
- what about unplanned subgroup or sensitivity analyses? I propose ...any subgroup or sensitivity analyses and whether they were preplanned.

- Agree if sources of bias is clearly explained.
- I totally agree and wording fine for a protocol. However, I wonder about the word "planned" for the actual report. Would authors interpret this as describe only planned analyses and ignore posthoc ones, including any requested by editors/peer reviewers?
- note the typo 'Sensitvity Analysis'
- "planned subgroup" and sensitivity analyses have different goals and should not be regrouped like this in an item. I agree with the need to describe them.
- This implies that users should not report unplanned analyses. Surely we would like researchers to report both and explicitly state which analyses were planned and which were unplanned?

Item 10a

This item is based on TRIPOD item 13a, which states: *"Describe the flow of participants through the study, including the number of participants with and without the outcome and, if applicable, a summary of the follow-up time. A diagram may be helpful"*.

Proposed checklist item (Delphi #1): Describe the flow of data sources and participants assessed and included, with reasons for exclusions. A diagram may be helpful.

Proposed checklist item (Delphi #2): Describe the flow of participants and data sources (assessed and included), including the number of participants with and without the outcome and, if applicable, a summary of the follow-up time. A diagram may be helpful.

Final Checklist item: Describe the number of clusters and participants from data identified through to data analysed. A flow chart may be helpful.

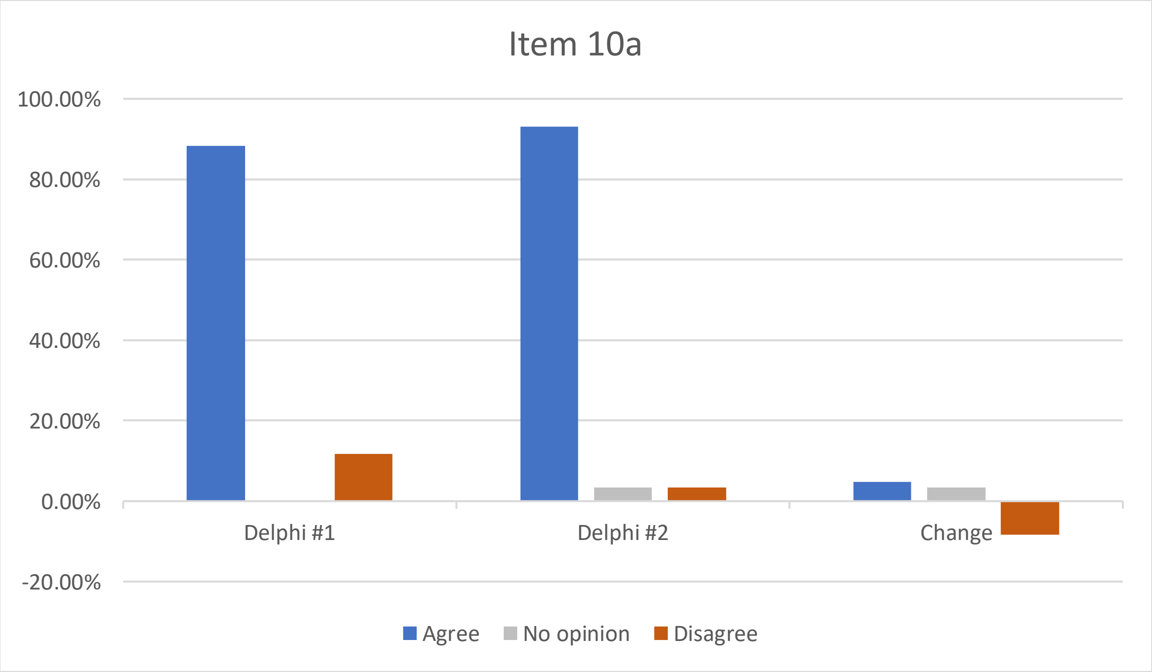

Figure 16 Results of the first Delphi round (N=17), the second Delphi round (N=29), and changes in agreement between the two rounds of the Delphi for item 10a ("Describe the number of clusters and participants from data identified through to data analysed. A flow chart may be helpful.")

Comments Delphi #1:

- Why have you left out information on the outcome?
- If there are several parts to the study e.g. development and validation perhaps of multiple models, in which different data sources are used for each then this can be tricky. Describe overall (combined data sources for all aspects) or separately for the different parts? Some guidance on this would be helpful.
- You removed specifying the number of participants with and without the outcome, but I believe this should be reported.
- Agree in principle but struggling to make sense of "flow of data sources"? Does this relate to 7c?
- The number of participants with and without the outcome and, if applicable, a summary of the follow-up time" should not be completely omitted from guidelines. Perhaps a separate item or part of item below clarification

Comments Delphi #2:

- I would say "describe the flow of data sources and participants".
- Not quite clear what 'assessed' means here. Is this all the available data that you begin with even if it wasn't all used?
- The item mentions flow of participants and the last sentence mentions a diagram. I would suggest replace diagram with flow chart. That covers also diagrams.
- Should we suggest more firmly the use of diagram?
- just a question: would you want to see the flow (/diagram) for each data source separately? I guess you would, right? This is currently not specifically mentioned in the item and may be something to be explicit about.

Item 10b

This new item is based on multiple items from the original TRIPOD checklist:

- Item 4a: *"Describe the study design or source of data (e.g., randomized trial, cohort, or registry data), separately for the development and validation data sets, if applicable"*
- Item 4b: *"Specify the key study dates, including start of accrual; end of accrual; and, if applicable, end of follow-up"*
- Item 5a: *"Specify key elements of the study setting (e.g., primary care, secondary care, general population) including number and location of centres"*
- Item 5c: *"Give details of treatments received, if relevant"*
- Item 13b: *"Describe the characteristics of the participants (basic demographics, clinical features, available predictors), including the number of participants with missing data for predictors and outcome"*
- Item 14a: *"Specify the number of participants and outcome events in each analysis"*

Proposed checklist item (Delphi #1): Report the main characteristics of the data for each study or setting, including the source, key study dates, case mix, sample size, and amount of missing data.

Proposed checklist item (Delphi #2): For the development data, report the characteristics of each data source or setting, including the key dates, predictors, treatments received, sample size, number of outcome events, and amount of missing data.

Final Checklist item: Report the characteristics overall and where applicable for each data source or setting, including the key dates, predictors, treatments received, sample size, number of outcome events, follow-up time, and amount of missing data.

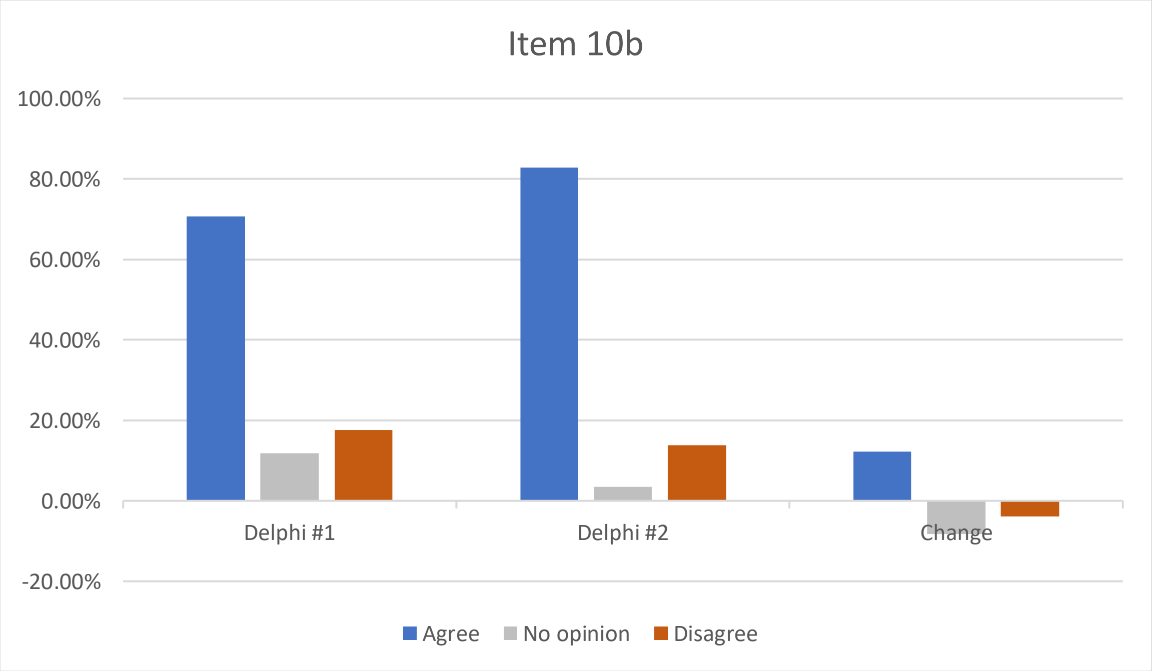

Figure 17 Results of the first Delphi round (N=17), the second Delphi round (N=29), and changes in agreement between the two rounds of the Delphi for item 10b ("Report the characteristics overall and where applicable for each data source or setting, including the key dates, predictors, treatments received, sample size, number of outcome events, follow-up time, and amount of missing data.")

Comments Delphi #1:

- Good to combine these!
- There are some pieces of the original items that are left out such as the distinction between development and validation sets and other examples. This new item may be less informative in that case. I suppose this will all be in the explanation document but there is a loss of information
- I would be interested in having the number of participants with and without the outcome reported (e.g. in the 'sample size' description).
- Perhaps some clarification on how would case-mix could be summarised and same for missing data as this could vary by predictor and data source. What about settings with many predictors and data sources? Also, whether treatment was given in some/all datasets should probably still be reported.
- There is no explicit mentioning of treatment and number of participants and outcome events, I believe these should be mentioned explicitly.
- How about key participant characteristics?
- As the focus thus far has been on data sources rather than simply studies, is it useful to include dataset in the list so it reads "...dataset, study or setting...".
- The phrase "for each study or setting" does not really make sense for individual registries but only really for IPD meta-analyses
- Number of events should be addressed. Type of missing data is important: structurally missing data will need to be addressed in linked data and EHR

Comments Delphi #2:

- What do we expect from an EHR type of study? Just one number (total dataset) for each of the characteristics or any subdivision of data?
- Applies to almost every item: what do you mean with 'every data source or setting'. Can the word setting be left out? Probably data from a different setting are also from a different source, by default?
- I find it nice to merge multiple previous items into this one.
- Would prefer more flexible language than the classic development - evaluation split. There are many other types of studies and analyses.
- Given that this mentions the need to report any treatment, I think one should also consider how treatment was handled in the model (e.g. to account for treatment 'drop-in')
- This item now asks for a lot of information, which may be confusing to those using TRIPOD and as a result may decrease the quality of reporting. Consider changing this item into multiple separate items.
- The individual items in TRIPOD provide illustrative examples. It would be helpful if these examples were to be found/linked to.

Item 10c

This item is based on TRIPOD item 13c, which states: *"For validation, show a comparison with the development data of the distribution of important variables (demographics, predictors and outcome"*.

Proposed checklist item (Delphi #1): For validation, show a comparison with the development data, including the source, key study dates, case mix, and sample size.

Proposed checklist item (Delphi #2): For the validation data, report the characteristics of each data source or setting, including the key dates, predictors, treatments received, sample size, number of outcome events, and amount of missing data. Show a comparison with the development data.

Actions: We revised the item text from the second Delphi round to focus on differences in patient characteristics (including the outcome) and to better distinguish between the reporting requirements from item 7c which states that *"For validation, identify any differences in definition and measurement from the development data (e.g., setting, eligibility criteria, outcome, predictors)"*.

Final Checklist item: For validation, show a comparison with the development data of the distribution of important variables (demographics, predictors, and outcome).

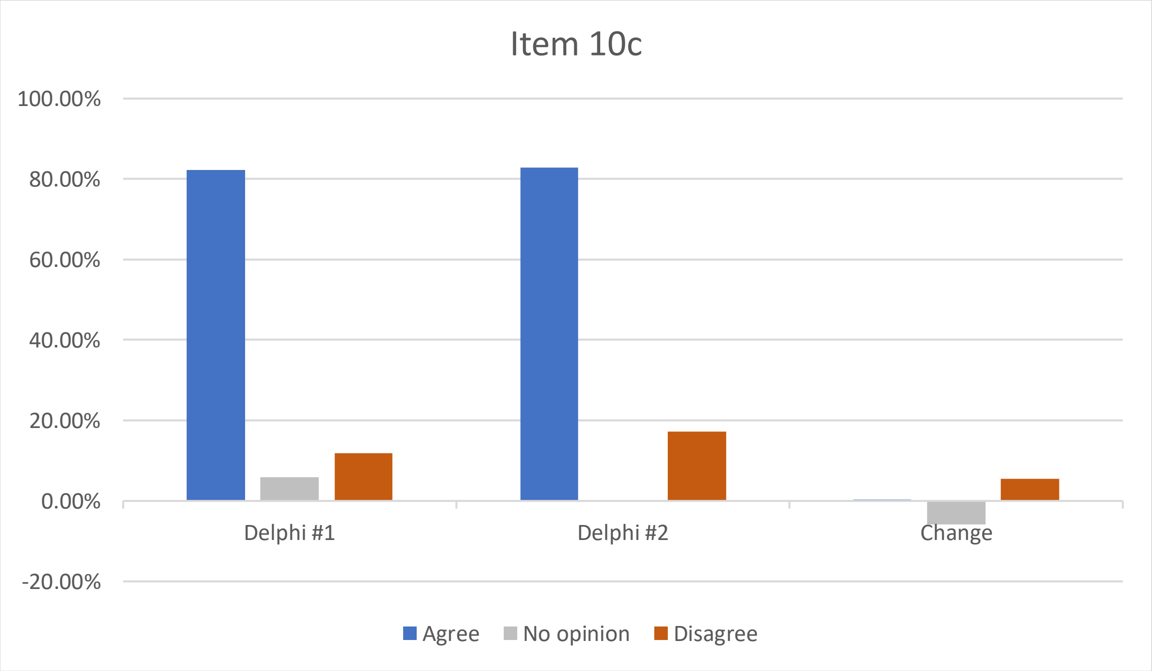

Figure 18 Results of the first Delphi round (N=17), the second Delphi round (N=29), and changes in agreement between the two rounds of the Delphi for item 10c ("For validation, show a comparison with the development data of the distribution of important variables (demographics, predictors, and outcome).")

Comments Delphi #1:

- Again, specific mention of outcome? Also: I've seen papers where they thought that this item required the use of statistical tests to compare dev and val! I think this should be discouraged.
- Much clearer
- Again, would be good to clarify how case mix could be compared, overall using linear predictor for example or for particular patient characteristics?
- I am not sure what is expected from the author here, so further clarification may be useful. Should the overall data be compared to the development data (which may or may not be a

big or IPD data set) --> for this the question sounds understandable. Or should each of the separate datasets (in case of IPD validation data) be compared to the development data? And if the development data is also IPD, then which comparison is expected, and how is this helpful to the reader?

- How about key participant characteristics?
- I don't understand what this is ask for.

Comments Delphi #2:

- I find it nice to merge multiple previous items into this one.
- See previous comment
- Show a comparison with the development data. what do you want to see? I wouldn't know what to do. Be more specific or delete it.
- As above
- Wording implies that only external validation is acceptable.
- see previous comment
- Likewise, the examples are missing to truly understand what is implied here.

Item 11

This is a new item that was not addressed by the original TRIPOD checklist.

Proposed checklist item (Delphi #1): Report the results from any risk of bias assessment (e.g. PROBAST) for each study or setting.

Proposed checklist item (Delphi #2): Report the results from any bias assessments (e.g. PROBAST), for each data source or setting.

Actions: We removed PROBAST from the item text because it is already suggested in item 7b which states that *"Describe the method for assessing risk of bias and applicability in the individual clusters (e.g., using PROBAST)"*. Furthermore, to better align with the terminology used by TRIPOD-Cluster, we simplified "study", "data source" and "setting" under the umbrella term "cluster".

Final Checklist item: Report the results of the risk of bias assessment in the individual clusters.

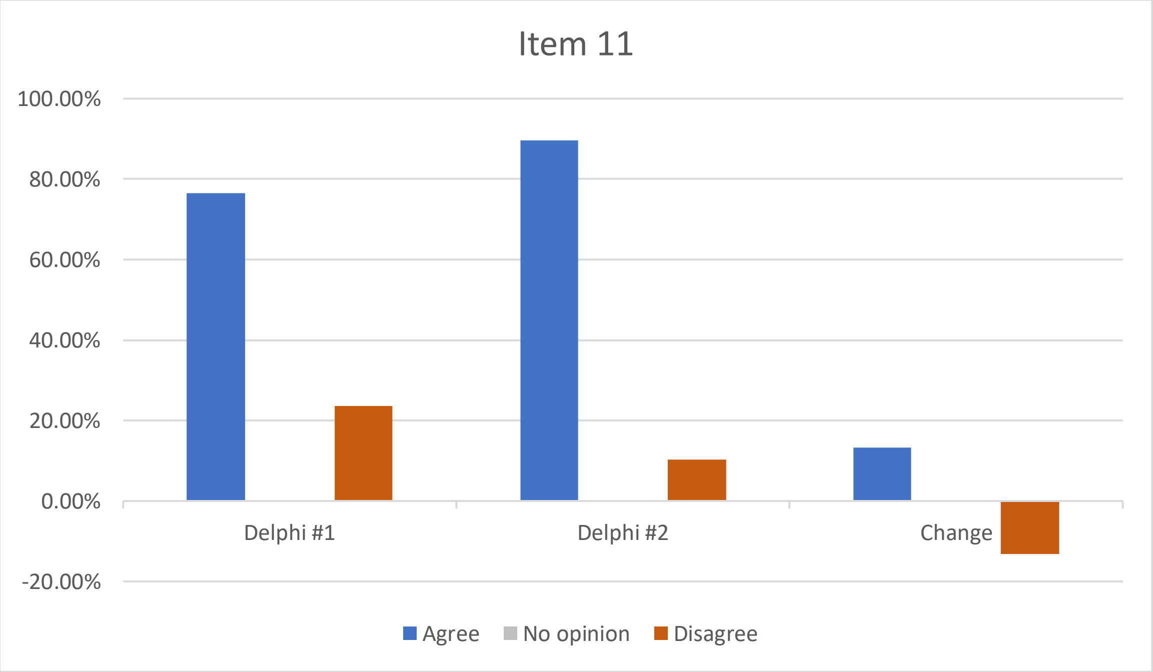

Figure 19 Results of the first Delphi round (N=17), the second Delphi round (N=29), and changes in agreement between the two rounds of the Delphi for item 11 ("Report the results of the risk of bias assessment in the individual clusters.").

Comments Delphi #1:

- As before this might be better done by independent researchers. But agree should be reported if appropriate.
- Previously referred to 'data source'
- Probably not relevant for non-IPD data (or otherwise at least unknown to broader big data
- audience)
- I think this is getting to be too much here. Too prescriptive
- See previous comment. The dataset may not be a study e.g. routine data collection. Or does
- setting address this?
- Again, this is really for IPD meta-analysis right?

Comments Delphi #2:

- See my previous comment regarding EHR type of studies.

- Risk of bias per data source can be reported in an appendix (together with scoring rules), however, the overall risk of bias per domain is preferably mentioned in the manuscript.
- "any risk of bias assessments"?
- Also, if any action was taken e.g. exclusions?
- PROBAST is quite extensive; if this needs to be done for each data source .. not so realistic?
- Same comment as above - better done as an independent assessment not by study authors.

Item 12a

This is a new item that was not addressed by the original TRIPOD checklist.

Proposed checklist item (Delphi #1): Report results of any heterogeneity in case-mix distributions and model parameters, and subsequent actions (e.g. omitting of predictors or datasets, or inclusion of dataset-specific terms in the model).

Proposed checklist item (Delphi #2): Report results of any heterogeneity (e.g., across data sources or settings) in model parameters, and subsequent actions (e.g., inclusion or exclusion of particular predictors or data sources).

Actions: We grouped the terms “data sources” , “settings” and “studies” into a single “cluster” term to avoid confusion and better align with the TRIPOD-Cluster terminology.

Final Checklist item: Report the results of any across-cluster heterogeneity assessments that led to subsequent actions during the model’s development (e.g., inclusion or exclusion of particular predictors or clusters).

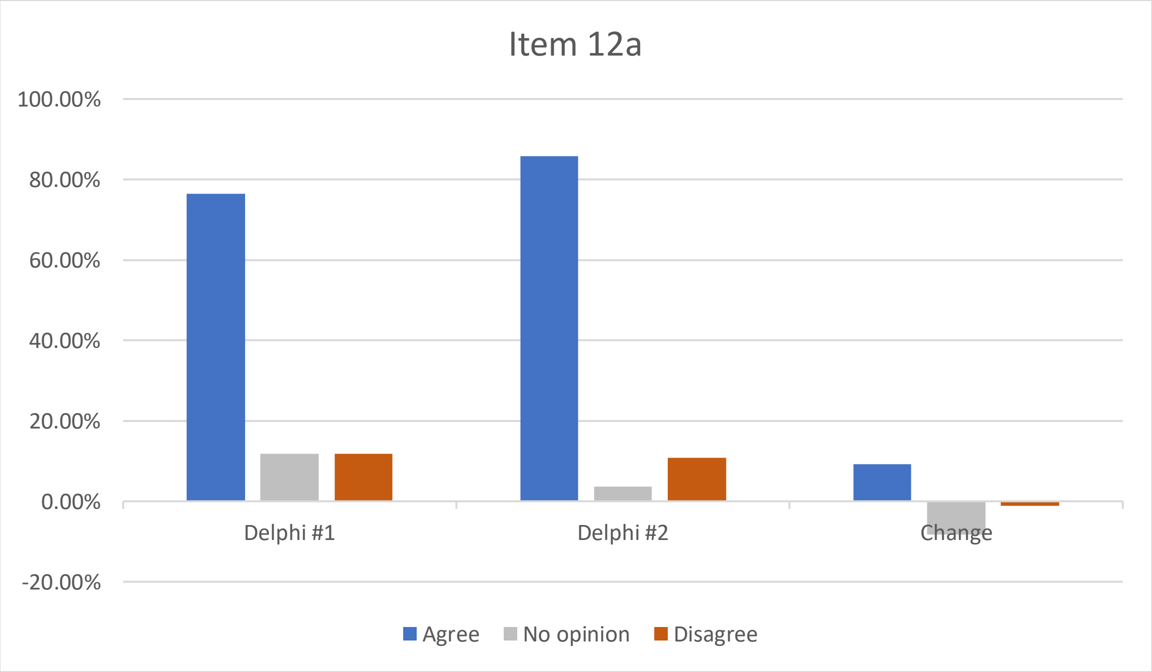

Figure 20 Results of the first Delphi round (N=17), the second Delphi round (N=28), and changes in agreement between the two rounds of the Delphi for item 12a (“Report the results of any across-cluster heterogeneity assessments that led to subsequent actions during the model’s development (e.g., inclusion or exclusion of particular predictors or clusters).”)

Comments Delphi #1:

- Does heterogeneity here refer to differences across data sources?
- 2 I would expect this to be pre-specified where possible in the methods section too.
- See my comment with the methods regarding this item
- You seem to have a big focus on case mix throughout. Many items are applicable to multiple data sources and data sets. Again, many newer models do not have specified predictors.
- What is "heterogeneity in model parameters"? I just found this confusing.

Comments Delphi #2:

- Too specific. There will be heterogeneity just from noise. The instruction are already very very specific and this is too much I think
- "Results of heterogeneity"?
- This is assuming that this is looked at in some depth. Might assume that model parameters are fixed (other than intercept). Might be that heterogeneity is explored more in terms of model performance rather than predictor effects.
- This is good - I like this much better than the other heterogeneity items I've seen so far
- This extends 8c; not necessary. Actions can be discussed with E&E on point 8c?
- Heterogeneity or differences between settings. These are not two distinctive phenomena. It should be more clear whether a difference between the two terms are implied or choose a single term.

Item 12b

This item is based on multiple TRIPOD items:

- Item 15a: *"Present the full prediction model to allow predictions for individuals (i.e., all regression coefficients, and model intercept or baseline survival at a given time point)"*
- Item 15b: *"Explain how to use the prediction model"*

Proposed checklist item (Delphi #1): Present the full prediction model (i.e. all model parameters) and explain how to use it for predictions in new individuals.

Proposed checklist item (Delphi #2): Present the full prediction model (i.e., all regression coefficients, and model intercept or baseline survival at a given time point) and explain how to use it for predictions in new individuals.

Final Checklist item: Present the final prediction model (i.e., all regression coefficients, and model intercept or baseline estimate of the outcome at a given time point) and explain how to use it for predictions in new individuals

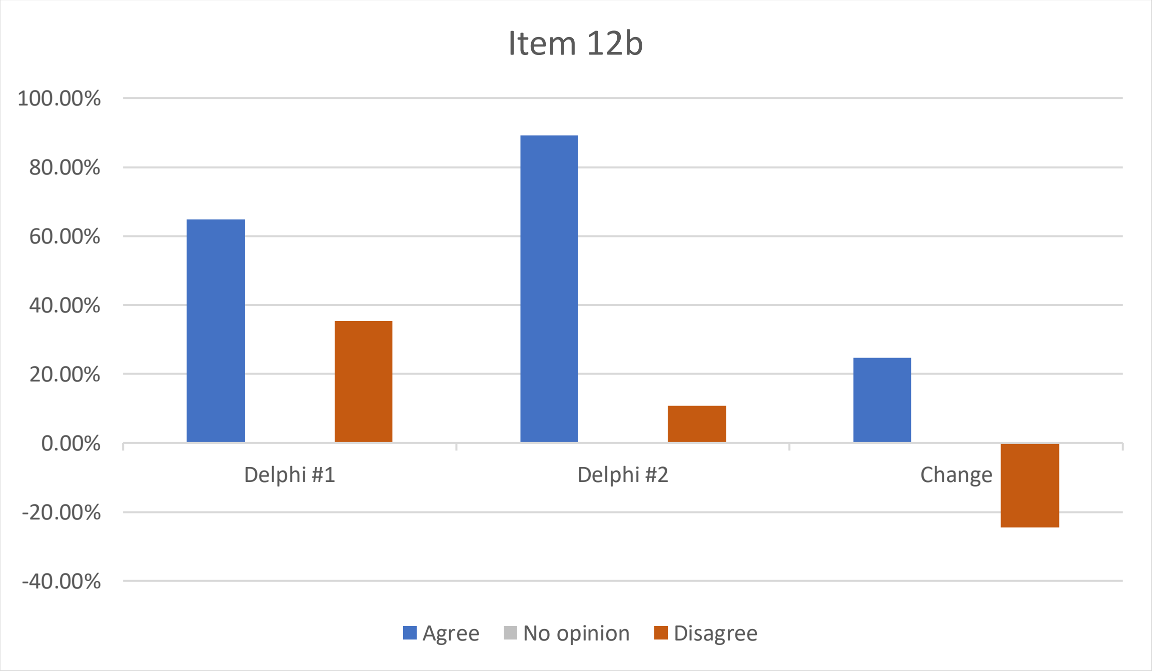

Figure 21 Results of the first Delphi round (N=17), the second Delphi round (N=28), and changes in agreement between the two rounds of the Delphi for item 12b ("Present the final prediction model (i.e., all regression coefficients, and model intercept or baseline estimate of the outcome at a given time point) and explain how to use it for predictions in new individuals.")

Comments Delphi #1:

- This should either be reported in the paper or information given on where this can be found. In some instances the model may be too complex for a full explanation in the text of a paper - e.g. AI models.
- I would specifically mention intercept/baseline survival in the item description.
- I would still mention examples of parameters that should be reported
- To include some description of how the model would be used in different settings if the model has been developed/updated in such a way? Links with earlier points about allowing for case-mix heterogeneity?

- Sometimes there is no model and no parameters, just an algorithm. It does need to be available to users, such as on a website.
- May new prediction models don't have an easily describable mean model. How is this to be done for a Neural net?
- And new datasets if dataset-specific terms are used.

Comments Delphi #2:

- How about more complex ML models?
- Might not want to limit to a single time point for baseline survival e.g. 'one or more time points'?
- I think this should recommend the reporting of baseline survival/hazard at ALL time points, rather than at an arbitrary given time point. The latter would allow users of the model to estimate absolute risks at any point in time and can be increasingly facilitated through techniques such as flexible parametric survival models.
- The model may be complex e.g. with numerous interaction terms or developed using machine learning approaches and there may be too many coefficients to present in full in a journal publication. Coefficients should be presented where feasible but I don't think this should be a requirement in all cases. Links to websites implementing the models should be encouraged.
- nice combo

Item 13a

This item is based on TRIPOD item 16, which states: *"Report performance measures (with CIs) for the prediction model"*.

Proposed checklist item (Delphi #1): Report prediction model performance (e.g. calibration and discrimination) and their precision, both overall and for each dataset or setting.

Proposed checklist item (Delphi #2): Report performance measures (with CIs) for the prediction model, overall and for each data source or setting.

Actions: We replaced confidence intervals into uncertainty intervals, since results can also be based on Bayesian analyses (which result into credibility intervals). Further, we grouped "source" and "setting" into a single term "cluster" to better align with the terminology of TRIPOD-Cluster.

Final Checklist item: Report performance measures (with uncertainty intervals) for the prediction model, overall and for each cluster.

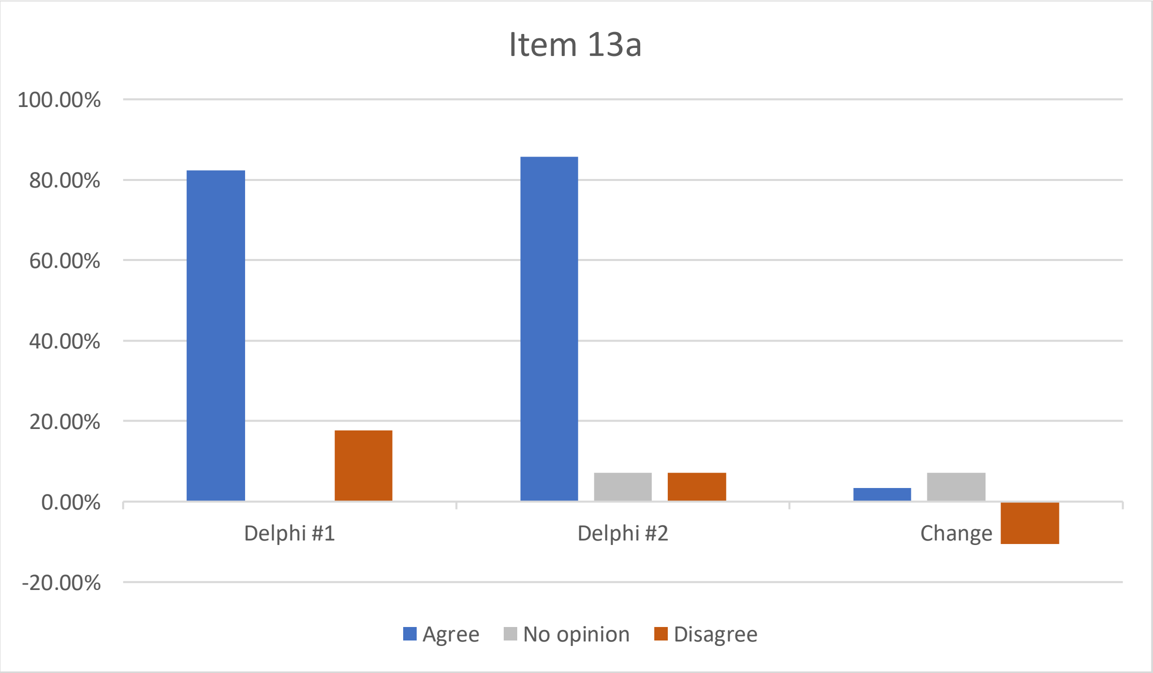

Figure 22 Results of the first Delphi round (N=17), the second Delphi round (N=28), and changes in agreement between the two rounds of the Delphi for item 13a ("Report performance measures (with uncertainty intervals) for the prediction model, overall and for each cluster.")

Comments Delphi #1:

- Keep as it was
- Maybe still mention CIs explicitly?
- And calibration across the risk spectrum?
- Maybe '... and their precision (e.g. with CIs), ...' to make it clear for those who would read it through?
- I would add 'where possible' as in some cases may have too few events to calculate performance statistics in some datasets or settings.
- I would add, if applicable after setting, as some big datasets do not consist of separate datasets or settings
- The old wording is more succinct and clear.

Comments Delphi #2:

- Again, studies having clustered data from one source (and setting) may not be triggered to do something
- For which situations is the overall performance measure still informative over the performance measures per data source or setting?
- might want to write CIs in full
- I would not use abbreviations.
- might need to say 'where possible' for each data source as this may not be possible or practical. E.g. if 100s/1000s of clusters, or if low event rate so some clusters wouldn't have events.
- Not sure if it is needed to report it for each study separately. The main interest is the overall performance.
- Would it be helpful to also give examples of performance measures to avoid too many reports of sensitivity and specificity rather than discrimination and calibration?
- suggest to add 'corrected for optimism'

Item 13b

This item was added after the second Delphi round to address results from item 8f (*"Describe how any heterogeneity across clusters (e.g., studies or settings) in model performance was handled and quantified."*)

Final Checklist item: Report results of any heterogeneity across clusters in model performance

Item 14

This item is based on TRIPOD item 17, which states: *"If done, report the results from any model updating (i.e., model specification, model performance)"*.

Proposed checklist item (Delphi #1): Report the results from any model updating (e.g. model parameters and performance) either overall or for particular datasets or settings.

Proposed checklist item (Delphi #2): Report the results from any model updating (including the updated model equation and subsequent performance), overall and for each data source or setting.

Final Checklist item: Report the results from any model updating (including the updated model equation and subsequent performance), overall and for each cluster.

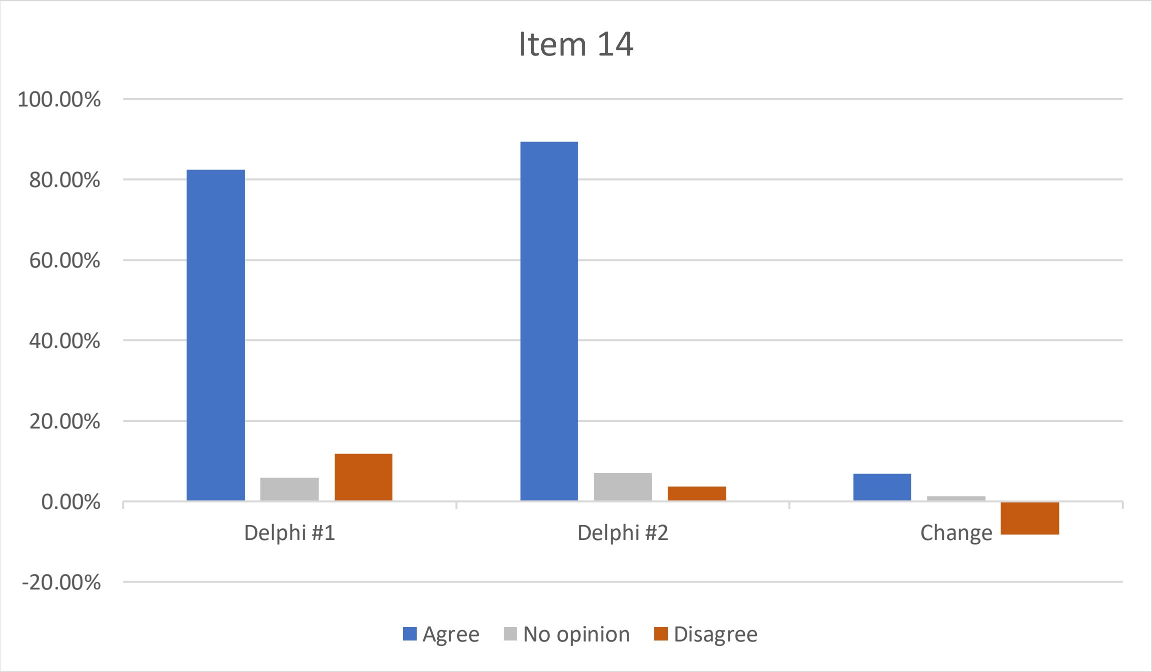

Figure 23 Results of the first Delphi round (N=17), the second Delphi round (N=28), and changes in agreement between the two rounds of the Delphi for item 14 ("Report the results from any model updating (including the updated model equation and subsequent performance), overall and for each cluster.").

Comments Delphi #1:

- don't understand why 'if done' is not necessary here
- What does model updating refer to? Updating for what?
- Including all model parameters so that it may be used in practice as proposed by the updating
- Again there might not be parameters.
- prefer old wording

Comments Delphi #2:

- See comments above. Model updating might only be interesting or relevant for a specific setting or subgroup.
- Same as previous point
- So long as there's a N/A box.
- Fine except ' and subsequent performance'. How do we know that?

Item 15

This new item was not included in the original TRIPOD checklist.

Proposed checklist item (Delphi #1): Report results from any subgroup or sensitivity analysis, e.g. performance according to risk of bias, participant characteristics, setting.

Proposed checklist item (Delphi #2): Report results from any subgroup or sensitivity analysis.

Final Checklist item: Report results from any subgroup or sensitivity analysis.

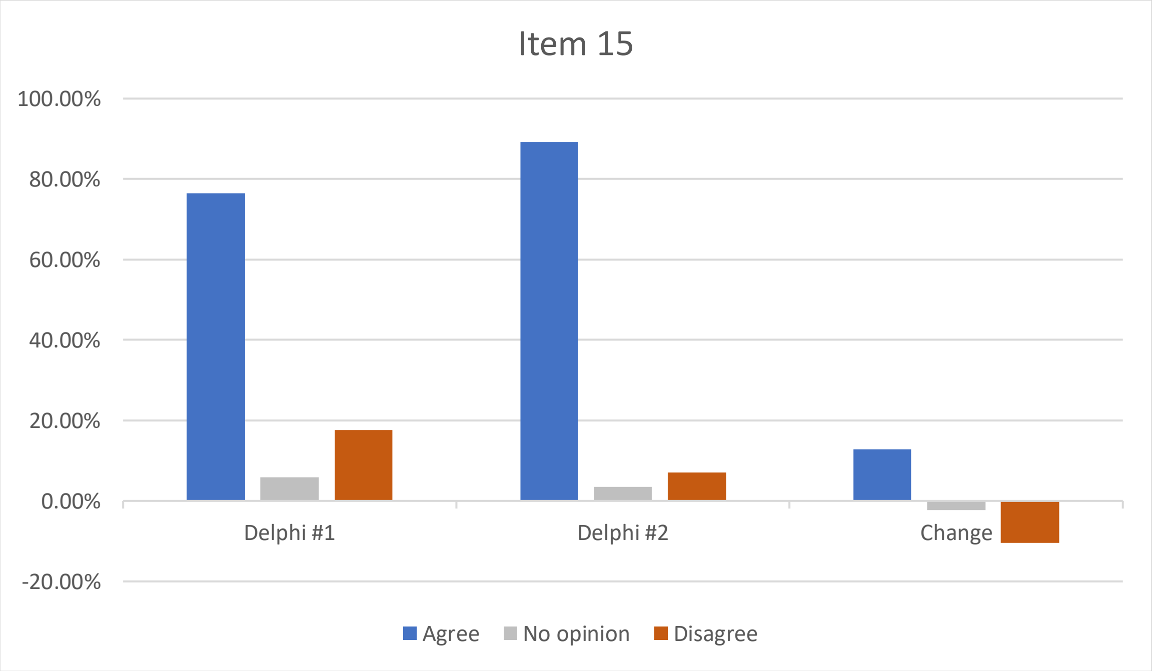

Figure 24 Results of the first Delphi round (N=17), the second Delphi round (N=28), and changes in agreement between the two rounds of the Delphi for item 15 ("Report results from any subgroup or sensitivity analysis.").

Comments Delphi #1:

- Subgrouping is a bad idea
- Too wordy. Just say "report results from relevant sensitivity analyses".
- Statement is ill-defined and subgroup analysis is dangerous.

Comments Delphi #2:

- A bit trivial
- This is a very general item and not very specific (that might be the intention).
- Wording implies that subgroup analysis is a good idea.
- Same comment as the previous question about subgroup and sensitivity.
- It would probably be good to give examples of what you consider to be "results". So does this relate to regression coefficients, model performance, etc.

Item 16a

This item is based on TRIPOD item 19b ("*Give an overall interpretation of the results, considering objectives, limitations, results from similar studies, and other relevant evidence*").

Proposed checklist item (Delphi #1): Give an overall interpretation of the main results, including heterogeneity in model performance, in the context of the objectives and previous studies.

Actions: We revised the item text to clarify that we are referring to heterogeneity across clusters. The strengths and limitations of the study are addressed in a separate reporting item 16c.

Final Checklist item: Give an overall interpretation of the main results, including heterogeneity across clusters in model performance, in the context of the objectives and previous studies.

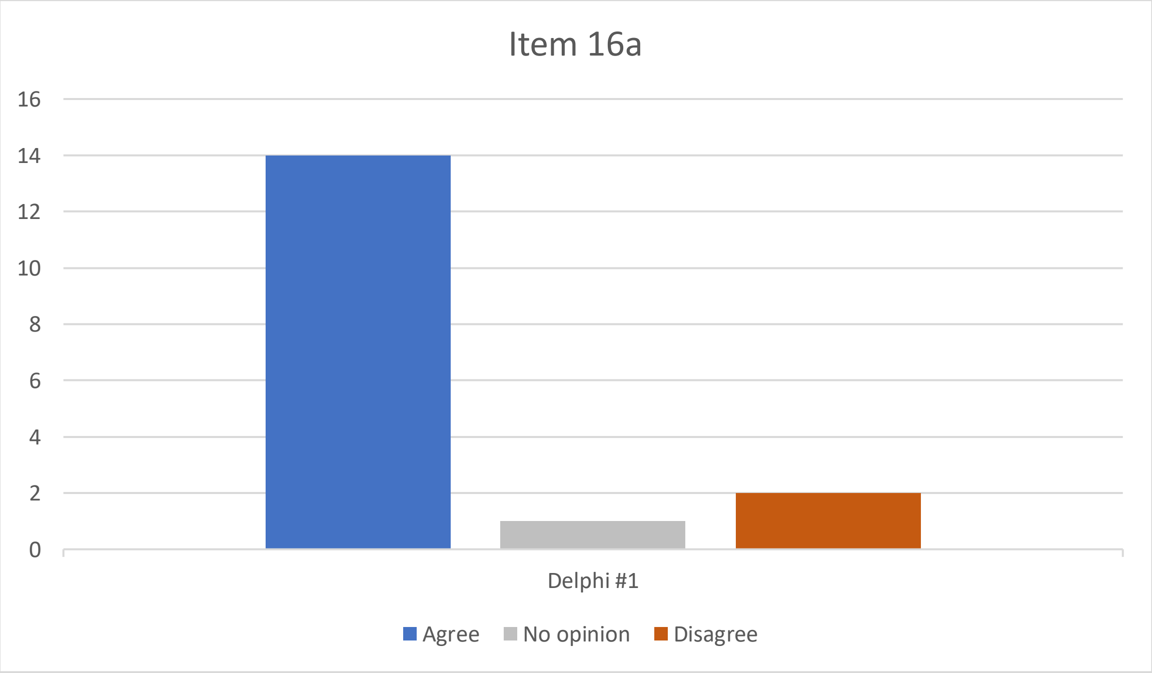

Figure 25 Results of the first Delphi round for item 16a ("Give an overall interpretation of the main results, including heterogeneity across clusters in model performance, in the context of the objectives and previous studies.").

Comments

- Again the term heterogeneity is vague
- I would not remove the word 'limitations' of the item, though. (The overall interpretation should also be done in the context of the limitations.)
- prefer original wording.

Item 16b

This item is based on TRIPOD item 19a ("*For validation, discuss the results with reference to performance in the development data, and any other validation data*").

Proposed checklist item (Delphi #1): For validation, discuss the results with reference to the model performance in the development data, and any previous validation data.

Actions: We made a minor change to the item text to clarify that authors should compare their results to previous validations of the same model in other data. We did not include this item in the second Delphi round because most participants agreed on its phrasing and relevance.

Final Checklist item: For validation, discuss the results with reference to the model performance in the development data, and in any previous validations.

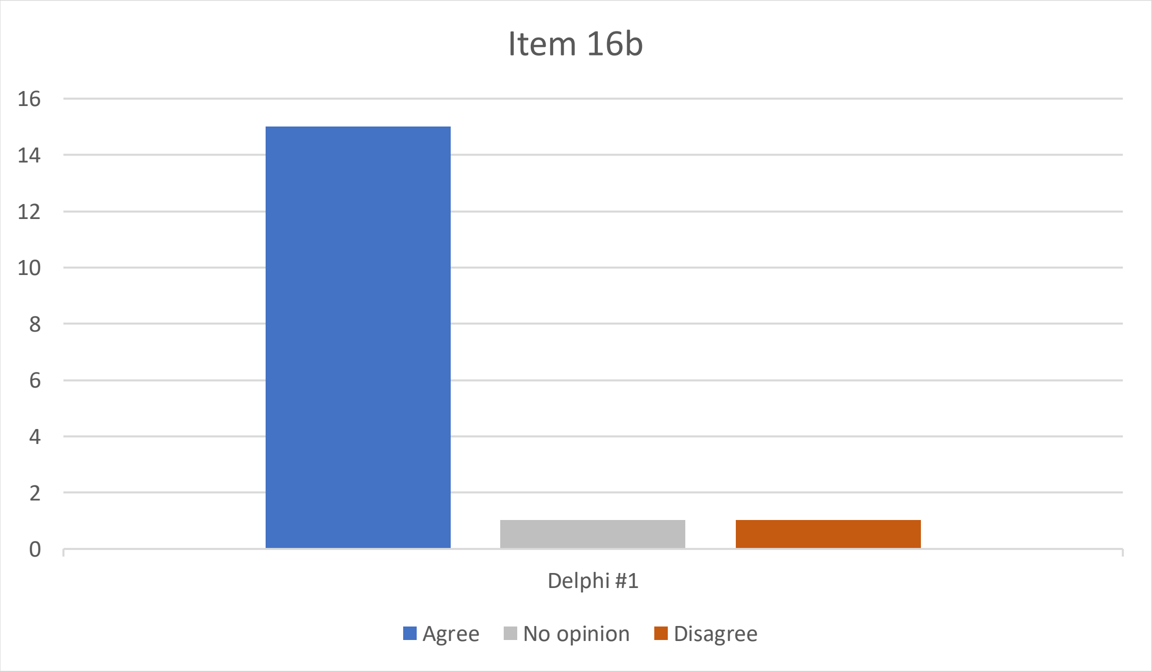

Figure 26 Results of the first Delphi round for item 16b ("For validation, discuss the results with reference to the model performance in the development data, and in any previous validations.").

Comments

- Not sure why the change.

Item 16c

This item is based on TRIPOD item 18, which states: "*Discuss any limitations of the study (such as nonrepresentative sample, few events per predictor, missing data)*".

Proposed checklist item (Delphi #1): Discuss the strengths of the study and any limitations (e.g. missing or incomplete data, risk of bias, data harmonization problems).

Actions: We changed the item text to include "representativeness of data sources", which was requested by several Delphi participants. We did not include this item in the second Delphi round because aforementioned issue was raised as main concern.

Final Checklist item: Discuss the strengths of the study and any limitations (e.g. missing or incomplete data, non-representativeness, data harmonisation problems).

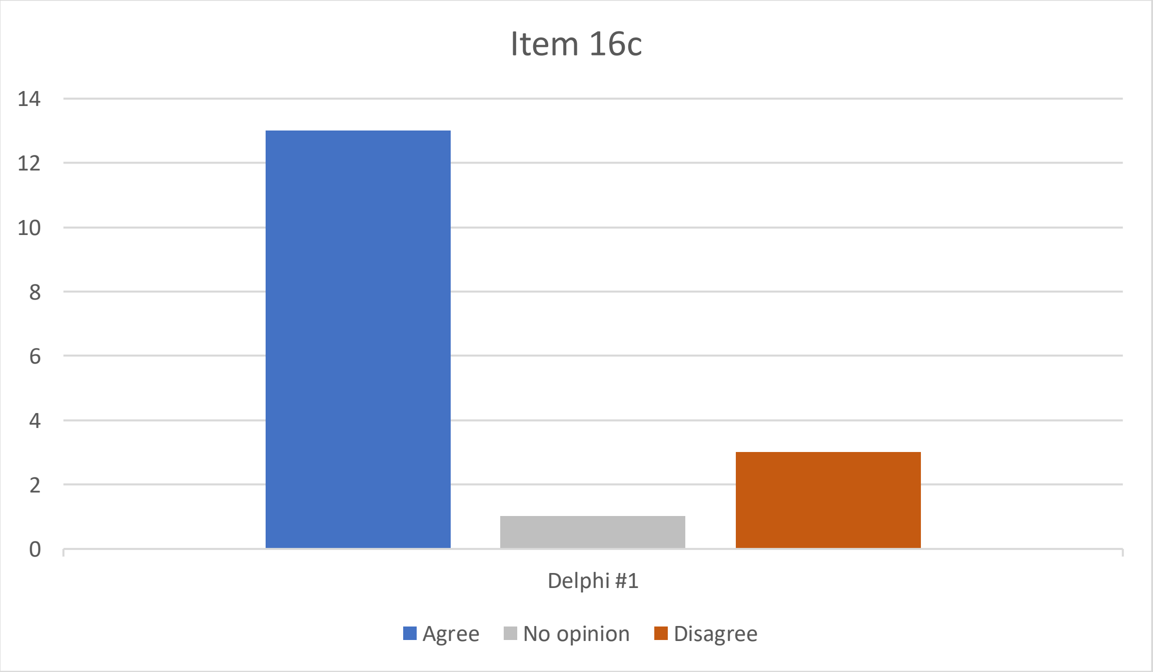

Figure 27 Results of the first Delphi round for item 16c ("Discuss the strengths of the study and any limitations (e.g. missing or incomplete data, non-representativeness, data harmonisation problems).").

Comments:

- Big Data are not necessarily with many events; and data hungry methods may have been used; so events per predictor (or another term) may still be relevant
- Add 'representativeness of data sources'?
- Sure!
- Are small sample size (outcomes) and nonrepresentativeness included in the risk of bias? If not, they should be discussed also.
- prefer old language
- representativeness remains crucial: combining data from multiple academic studies conducted in specialist fields does not guarantee applicability in general care

Item 17

This item is based on TRIPOD item 20, which states: *"Discuss the potential clinical use of the model and implications for future research"*.

Proposed checklist item (Delphi #1): Discuss the potential clinical use of the model and implications for future research, with specific view to generalizability and applicability of the model across different settings or (sub)populations.

Actions: No further item changes were made after the first Delphi round, since most participants agreed on its phrasing and relevance.

Final Checklist item: Discuss the potential use of the model and implications for future research, with specific view to generalizability and applicability of the model across different settings or (sub)populations.

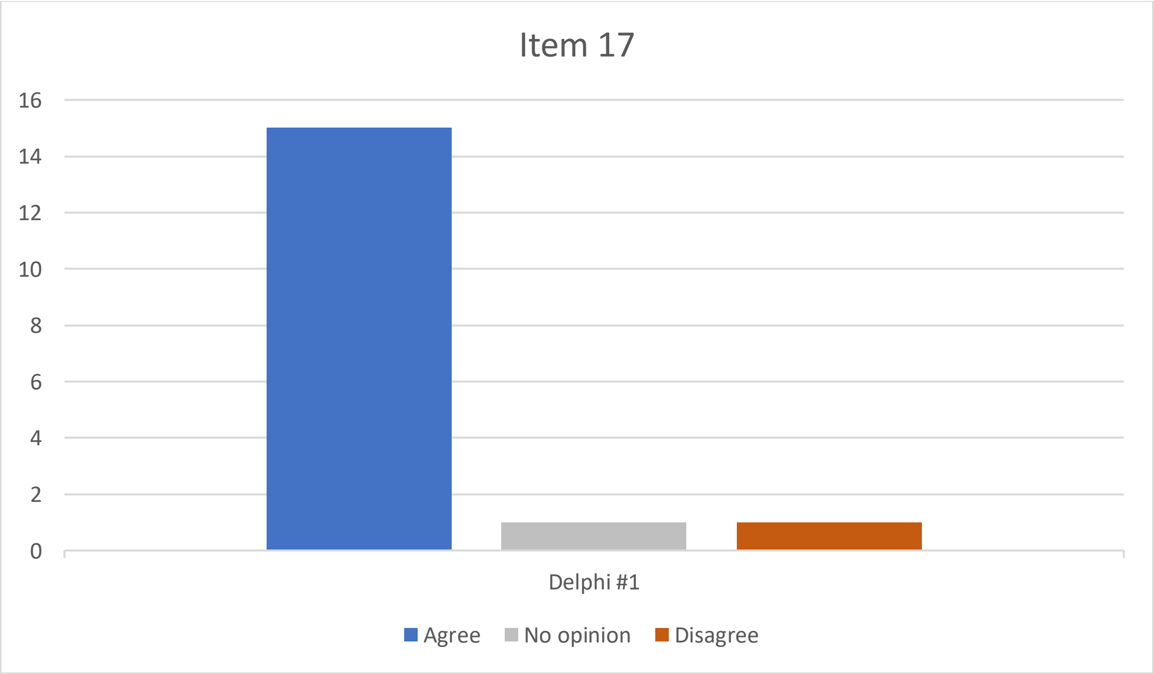

Figure 28 Results of the first Delphi round for item 17 ("Discuss the potential use of the model and implications for future research, with specific view to generalizability and applicability of the model across different settings or (sub)populations.").

Comments:

- Yes!
- prefer old wording

Item 18

The text for this item is (nearly) identical to item 21 from the original TRIPOD checklist, which states: *"Provide information about the availability of supplementary resources, such as study protocol, Web calculator, and datasets"*.

Final Checklist item: Provide information about the availability of supplementary resources (e.g., study protocol, analysis code, data sets).

Item 19

The text for this item is identical to item 22from the original TRIPOD checklist, which states: *"Give the source of funding and the role of the funders for the present study"*.

Final Checklist item: Give the source of funding and the role of the funders for the present study.
